# Conserved allosteric inhibitory site on the respiratory syncytial virus and human metapneumovirus RNA-dependent RNA polymerases

**DOI:** 10.1038/s42003-023-04990-0

**Published:** 2023-06-19

**Authors:** Victoria A. Kleiner, Thierry O. Fischmann, John A. Howe, Douglas C. Beshore, Michael J. Eddins, Yan Hou, Todd Mayhood, Daniel Klein, Debbie D. Nahas, Bob J. Lucas, He Xi, Edward Murray, Daphne Y. Ma, Krista Getty, Rachel Fearns

**Affiliations:** 1 Department of Virology, Immunology & Microbiology, National Emerging Infectious Diseases Laboratories, Boston University Chobanian and Avedisian School of Medicine, Boston, MA USA; 2 MRL, Merck & Co., Inc., Rahway, NJ USA

**Keywords:** Cryoelectron microscopy, Antivirals, Virus structures

## Abstract

Respiratory syncytial virus (RSV) and human metapneumovirus (HMPV) are related RNA viruses responsible for severe respiratory infections and resulting disease in infants, elderly, and immunocompromised adults^1–3^. Therapeutic small molecule inhibitors that bind to the RSV polymerase and inhibit viral replication are being developed, but their binding sites and molecular mechanisms of action remain largely unknown^4^. Here we report a conserved allosteric inhibitory site identified on the L polymerase proteins of RSV and HMPV that can be targeted by a dual-specificity, non-nucleoside inhibitor, termed MRK-1. Cryo-EM structures of the inhibitor in complexes with truncated RSV and full-length HMPV polymerase proteins provide a structural understanding of how MRK-1 is active against both viruses. Functional analyses indicate that MRK-1 inhibits conformational changes necessary for the polymerase to engage in RNA synthesis initiation and to transition into an elongation mode. Competition studies reveal that the MRK-1 binding pocket is distinct from that of a capping inhibitor with an overlapping resistance profile, suggesting that the polymerase conformation bound by MRK-1 may be distinct from that involved in mRNA capping. These findings should facilitate optimization of dual RSV and HMPV replication inhibitors and provide insights into the molecular mechanisms underlying their polymerase activities.

## Abstract (web summary)

A conserved allosteric site identified on the L polymerase proteins of RSV and HMPV can be targeted by a dual-specificity, non-nucleoside inhibitor that acts by inhibiting conformational changes necessary for RNA synthesis initiation and elongation.

## Introduction

RSV and HMPV are significant causes of respiratory disease, with the capacity to cause severe bronchiolitis, pneumonia, and death, particularly in infants, young children, and the elderly. For example, systematic review studies have estimated that, annually, RSV causes over 100,000 deaths in children younger than 5 years^5,6^ and 14,000 in-hospital deaths in individuals over 65 years of age^7^. While the global impact of HMPV is less well characterized, this virus also inflicts a significant disease burden^3,8,9^. RSV and HMPV are within the family *Pneumoviridae* of the order *Mononegavirales*, the non-segmented, negative-strand RNA viruses (nsNSVs)^10^. Although RSV and HMPV have many similarities, they differ in their genome organization, number of genes, and expressed proteins, and they are classified in separate genera^9–11^.

An attractive target for development of pneumovirus antivirals is the viral polymerase^4^. The polymerase is a multifunctional enzyme that is responsible for transcribing the viral negative sense RNA genome to generate polyadenylated mRNAs with a 5′ methylguanosine cap and replicating it to generate new viral genomes^12,13^. The polymerase is comprised of a complex of a viral large polymerase subunit protein (L) monomer and phosphoprotein (P) tetramer^14–16^. The L protein contains three enzymatic domains: an RNA-dependent RNA polymerase (RdRp), a polyribonucleotidyltransferase (PRNTase), which is involved in cap addition, and a methyltransferase (MTase) that methylates the cap. In addition, it contains a connector domain (CD) and C-terminal domain (CTD) that are thought to facilitate domain rearrangement and buttress the MTase domain, respectively^14,17,18^. Cryo-EM structures for the RSV and HMPV polymerases and polymerases of three related viral families, the rhabdoviruses, vesicular stomatitis virus (VSV) and rabies virus; the paramyxoviruses, parainfluenza virus type 5 (PIV-5), Newcastle disease virus (NDV), and parainfluenza virus type 3 (PIV-3); and the filovirus Ebola virus (EBOV); show that the polymerases have shared structural features, but different arrangements of some domains and motifs^14–16,19–25^. These differences are thought to reflect distinct conformational states that the polymerase adopts at different stages of transcription and RNA replication^26–28^.

Both the functional domains and structural features that allow conformational transitions have the potential to be targeted with non-nucleoside analog inhibitors (NNIs). Several NNIs of the RSV L protein have been described and their mechanisms of inhibition characterized. YM-53403 and its derivatives, AZ-27 and PC-786, elicit resistance in a linker region between the CD and MTase domains^29–32^, with AZ-27 inhibiting initiation of RNA synthesis at the promoter^30^. An AVG series of compounds elicit resistance in the same linker region and in the CD^30,33,34^. These compounds bind to an interface between the PRNTase, CD, and MTase domains, as demonstrated by photoaffinity cross-linking, and inhibit the polymerase from elongating the RNA^30,33,34^. Finally, the BI compound series elicits resistance in the PRNTase domain, and compound BI-D inhibits cap addition and causes dysregulated elongation^35,36^. Other classes of replication inhibitors exist, but their mechanisms of inhibition have not been well characterized^4^.

The large size of the nsNSV polymerases, coupled with their multifunctional nature, makes them challenging to study. In addition to having potential for antiviral drug development, inhibitors can be valuable tool compounds that provide insight into how polymerase structure relates to its different functional activities. In this study, we analyzed the interaction between a non-nucleoside pneumovirus inhibitor, referred to as MRK-1, and the RSV and HMPV polymerases. Cryo-EM structures of MRK-1 liganded polymerase complexes revealed the pocket on each of the polymerases to which MRK-1 binds. Functional analysis of the RSV polymerase in the presence of MRK-1 showed that the compound inhibits both initiation and early elongation, indicating that it functions as an allosteric inhibitor that prevents conformational changes that the polymerase undergoes during the early stages of RNA synthesis.

## Results

### MRK-1 has antiviral activity against RSV and HMPV

**Fig. 1:**
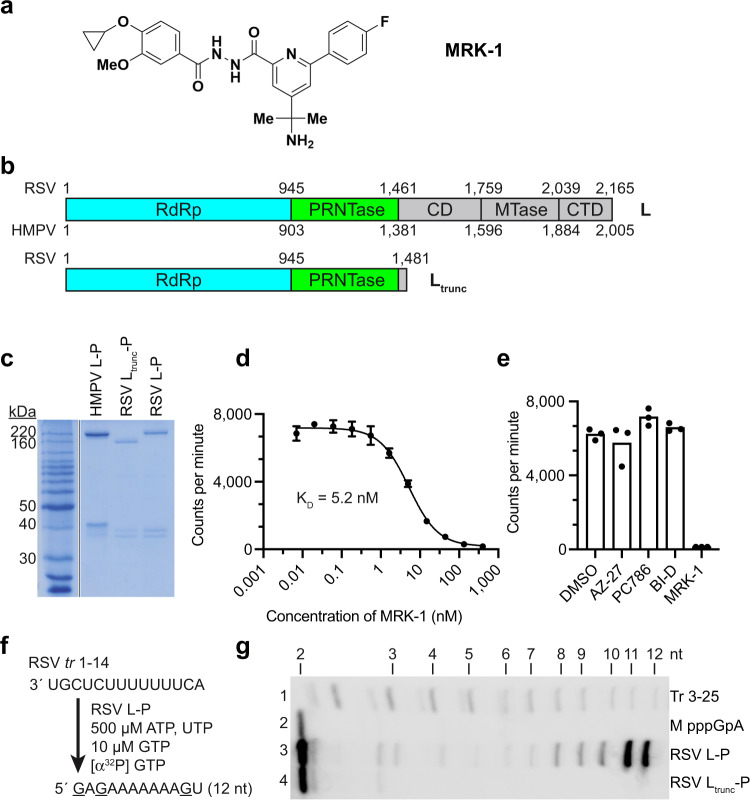
Characterization of purified L proteins. **a** Chemical structure of MRK-1. **b** Schematic diagram illustrating the domain organizations of full-length RSV and HMPV L proteins and truncated RSV L protein (L_trunc_). Flexible domains not captured in pneumovirus L structures are colored in gray. **c** SDS-PAGE of purified L-P and L_trunc_-P proteins migrated alongside a BenchMark protein ladder (Invitrogen). **d** Titration of unlabeled MRK-1 against radiolabeled MRK-1 to determine the affinity of binding to wild-type (WT) RSV L-P. The data show the mean and data points for three technical replicates, representative of one of three independent experiments (see also Supplementary Fig. 1a, c). Dissociation constant (K_D_) is denoted under the curve. **e** Analysis of competition between unlabeled NNIs and radiolabeled MRK-1 for binding to WT RSV L-P. Data show the mean and data points for three technical replicates, representative of one of three biological replicate experiments (see also Supplementary Fig. 1b, d). **f** Schematic diagram illustrating the design of the RNA synthesis assay. **g** RNA synthesis activities of RSV L-P and L_trunc_-P from position 3C of the RSV *trailer* (*tr*) promoter. A phosphorimage representing one of three independent experiments is shown. Lane 1 shows a ladder representing products generated from position 3C of a *trailer* 1-25 promoter. Note that the ladder RNAs have a 5′ monophosphate and products ≤6 nt in length migrate differently than RNA with a 5′ triphosphate. Lane 2 shows a marker for the pppGpA dinucleotide.

Previously, a class of pneumovirus NNIs was described^4^. Based on these findings, we generated a related NNI, termed MRK-1 (Fig. 1a, Supplementary Note 1). The antiviral activity of MRK-1 against RSV and HMPV was evaluated in cell-based virus replication assays (Supplementary Table 1). MRK-1 was active against RSV-A2/EGFP (EC_50_ = 2.1 nM) in a GFP reporter assay, the lab-adapted strains RSV-A Long (EC_50_ = 3.9 nM) and RSV-B Washington in plaque reduction assays (EC_50_ = 3.3 nM), and the clinical isolates RSV-A and RSV-B using cytopathic effect assays (EC_50_ = 4.3 nM and EC_50_ = 1.3 nM, respectively). MRK-1 was also active against HMPV/EGFP (EC_50_ = 185 nM), albeit with reduced potency. Resistance profiling identified that the mutation L I1381T, which lies in the PRNTase domain (Fig. 1b, Supplementary Table 1), conferred partial resistance to MRK-1, providing confirmatory evidence that MRK-1 targets that pneumovirus polymerases.

### MRK-1 binds to a distinct pocket from other known RSV polymerase inhibitors

To characterize the interaction between RSV L and MRK-1, the full-length RSV L-P complex was purified (Fig. 1b and c) and a radioligand binding assay was used to determine the affinity of MRK-1 binding with ^3^H-labeled MRK-1 (synthesized as described in Supplementary Note 1). By titrating unlabeled MRK-1 against complexed ^3^H-labeled MRK-1, it was determined that MRK-1 interacts with RSV L-P with dissociation constants (K_D_) ranging from 2.3 to 5.2 nM, depending on the experimental conditions (Fig. 1d, Supplementary Fig. 1a, c). Competition assays were performed with other RSV L NNIs. This analysis showed that ^3^H-MRK-1 binding was inhibited by preincubation of the RSV L-P complex with unlabeled MRK-1, but not with the RSV NNIs AZ-27, PC-786, or BI-D (Fig. 1e, Supplementary Fig. 1b, d). This finding indicates that MRK-1 binds to a distinct pocket from other known RSV inhibitors.

### A cryo-EM structure reveals the binding pocket of MRK-1 on RSV L

**Fig. 2:**
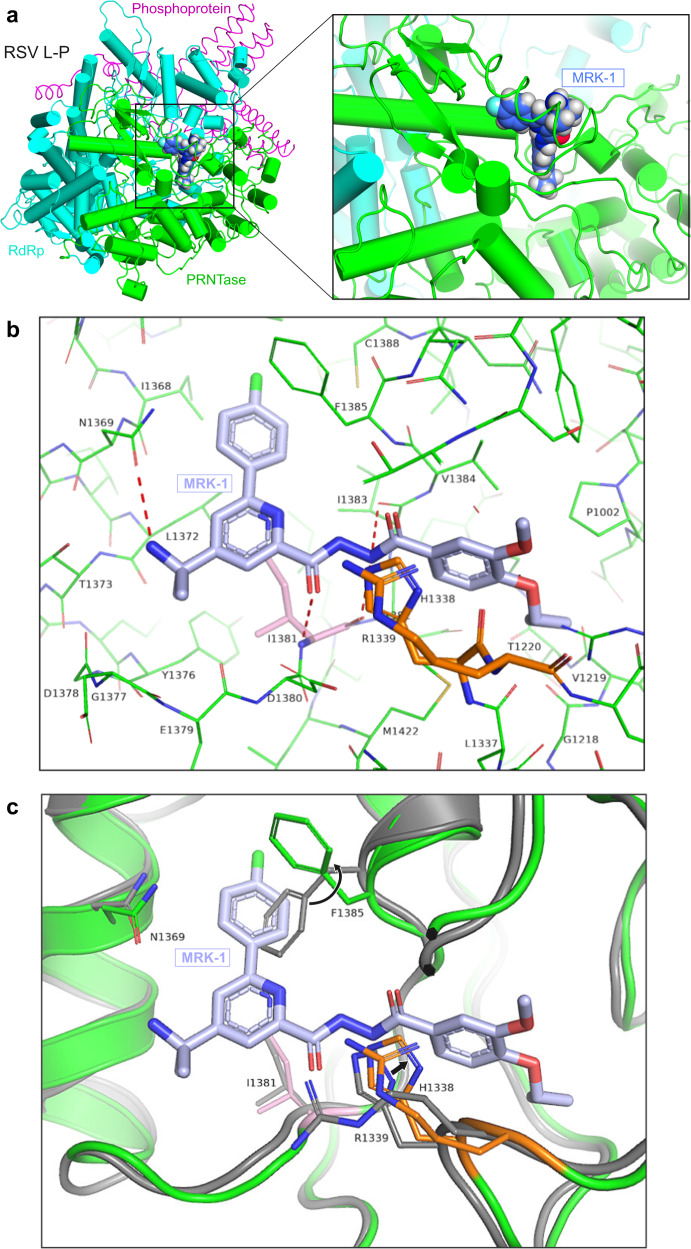
Structure of RSV L in complex with MRK-1. **a** The structure of RSV L_trunc_-P in complex with the MRK-1 ligand (PDB ID: 8FPI). The RdRp and PRNTase domains are in blue and green, respectively, and the P tetramer is in pink. A magnified view of MRK-1 and its binding pocket are shown. **b** Magnified view of the MRK-1 binding pocket with L side chains shown as green sticks. Ile1381 is shown in pink and the HR motif in orange. **c** Comparison of the binding site between MRK-1 bound and *apo* RSV polymerase structures (PDB IDs: 8FPI and 6PZK, respectively). The MRK-1 liganded structure is superimposed on the *apo* L structure. The *apo* RSV L structure is shown in gray, with the MRK-1 bound structure shown in green. Rearrangements are indicated with black arrows.

To date, no structure of liganded pneumovirus L protein has been disclosed. Initial attempts to solve MRK-1 liganded cryo-EM structures with full-length RSV L protein yielded low-resolution structures. Based on previous reports showing that the RSV CD, MTase, and CTD were disordered^14,15^, a reductionist strategy was devised in which the L protein was truncated to remove these domains, with the rationale that their removal might aid particle density and orientations. The remaining N-terminal-RdRp-PRNTase core construct (termed L_trunc_; Fig. 1b) yielded soluble, stable protein (Fig. 1c) that was functional in initiation of RNA synthesis, but defective in elongation (Fig. 1f, g), reminiscent of an RSV L construct truncated at the C-terminal end of the CD^34^. L_trunc_-P was readily amenable to addition of the MRK-1 ligand and a co-complex structure was solved at a resolution of 2.39 Å (Table 1, Supplementary Fig. 2). The RdRp and PRNTase domains of L and tetrameric P protein were resolved in the structure, which exhibits the same overall architecture as the *apo* polymerase (average r.m.s.d. 0.49 Å over 1305 residue Cα’s) (Fig. 2a, Supplementary Table 2). This result confirms that the CD, MTase, and CTD of L can be removed without impacting the remaining structure of the L-P complex, consistent with prior observations that these domains are highly flexible in the *apo* state of full-length L-P. As observed in prior unliganded structures of RSV polymerase, the MRK-1 liganded structure represents the polymerase in a non-initiation state with the priming loop folded into the PRNTase domain and an open channel between the RdRp and PRNTase domains that might be required for transit of the nascent RNA to the PRNTase domain, as suggested previously^14,15^.

**Table 1:** Cryo-EM data collection, refinement, and validation statistics.

	RSV L_trunc_-P in complex with MRK-1 (EMDB-29365) (PDB 8FPI)	HMPV L-P in complex with MRK-1 (EMDB-29366) (PDB 8FPJ)
*Data collection and processing*		
Magnification		
Voltage (kV)	300	300
Electron exposure (e^–^/Å^2^)	34.5	45.0
Defocus range (μm)	−0.8 to −2.2 µm	−0.8 to −2.2 µm
Pixel size (Å)	1.06	0.815
Symmetry imposed	C1	C1
Initial particle images (no.)	1,540,913	5,087,965
Final particle images (no.)	589,457	524,654
Map resolution (Å)	2.39	2.74
FSC threshold	0.143	0.143
*Refinement*		
Initial model used (PDB code)	6PZK	6U5O
Model composition		
Non-hydrogen atoms	8222	7963
Protein residues	1619	1596
Ligands	35	35
*B* factors (Å^2^)		
Protein	53.2	43.1
Ligand	53.4	38.5
R.m.s. deviations		
Bond lengths (Å)	0.008	0.003
Bond angles (°)	0.885	0.569
Validation		
MolProbity score	2.53	1.71
Clashscore	11.8	5.7
Poor rotamers (%)	5.65	0.00
Ramachandran plot		
Favored (%)	94.88	94.30
Allowed (%)	4.93	5.70
Disallowed (%)	0.19	0.00

Density corresponding to MRK-1 was identified in a pocket located within the PRNTase domain and formed by the conserved motifs B, D, and E (Fig. 2a, b, Supplementary Fig. 3). There are few conformational differences in the PRNTase pocket between the *apo* and MRK-1 bound structures of RSV L (Fig. 2c). Phe1385 (motif E) undergoes a rotamer change to accommodate the fluorophenyl group of MRK-1 and make an edge-face pi interaction. The side chain of His1338 (motif D) also rotates nearly 90˚ to accommodate MRK-1. There are several key interactions between the ligand and RSV L. The hydrazide of MRK-1 forms hydrogen bonds with the backbone carbonyl of Ile1381 and the side chain of His1338. The 4-cyclopropoxy-3-methoxybenzoyl group lies on Val1384, while also contacting Pro1002, Gly1218 (motif A), and Arg1345. At the other end, the fluorophenyl group of MRK-1 sits in a hydrophobic pocket created by Ile1241, Ile1368, and Thr1365. The site of partial resistance to MRK-1, Ile1381 (Supplementary Table 1), contacts the central pyridinyl ring of MRK-1 (Fig. 2b). Presumably mutation of this residue to Thr perturbs the binding of MRK-1 by changing the polarity of the pocket since the structure suggests that the mutation would not cause a steric clash with the inhibitor. Perhaps the most surprising aspect of the inhibitor-bound structure is how little of the protein is changed relative to the *apo* state (Fig. 2c). The MRK-1 pocket is largely pre-formed except for the rotation of Phe1385, indicating that inhibitors of this class may target a low energy state of the polymerase without induced fit.

### The HMPV L protein has a similar, but not identical, binding pocket for MRK-1

**Fig. 3:**
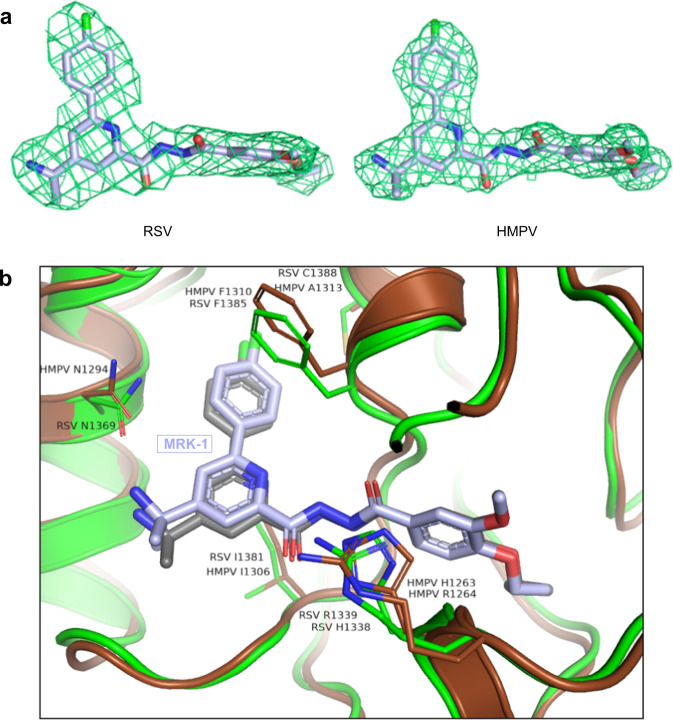
Comparison of MRK-1 binding in RSV and HMPV polymerases. **a** Aligned electron density maps of MRK-1 bound in the RSV and HMPV polymerases (PDB IDs: 8FPI and 8FPJ, respectively). **b** Comparison of the binding site between MRK-1 bound RSV (protein side chains in green, compound in gray) and HMPV (protein side chains in brown, compound in light blue) L structures (PDB IDs: 8FPI and 8FPJ, respectively). Labeled residues have undergone similar structural rearrangement in both RSV and HMPV L proteins upon MRK-1 binding.

As noted above, MRK-1 has potency against HMPV in addition to RSV (Supplementary Table 1). To compare the binding of MRK-1 to HMPV L with that of RSV L, full-length HMPV L-P complexes were purified (Fig. 1a, c) and the structure of the MRK-1 liganded HMPV L-P structure was solved at a resolution of 2.74 Å (Table 1, Supplementary Figs. 2 and 4a). The structure of MRK-1-bound HMPV “L” chain Cα backbone superposes over that of the *apo* enzyme with a r.m.s.d. of 1.1 Å for 1204 atoms, indicating that the structures are similar (Supplementary Fig. 4b, Supplementary Table 2). The inhibitor binds in the groove present on the PRNTase domain next to the catalytic His1263 residue, equivalent to the RSV binding site, mimicking the same binding pose (Fig. 3a, b). In addition, the HMPV polymerase structure undergoes similar local structural rearrangements upon complexation with the ligand, namely re-orientation of the HMPV L His1263, Arg1264, and Phe1310 side chains that correspond to RSV L His1338, Arg1339, and Phe1385 (Supplementary Fig. 4b). Hence the MRK-1-liganded RSV and HMPV structures are highly similar with each other, explaining why MRK-1 has antiviral activities against both viruses.

The observed potencies of MRK-1 against the RSV and HMPV polymerases differ between the two viral strains by approximately two orders of magnitude (Supplementary Table 1), which may in part reflect differences in assays. However, a structural rationale for these differences may be based on the lone sequence mismatch among amino acids with their side chains pointing toward the ligand between the polymerases’ MRK-1 binding sites; specifically, RSV Cys1388 analogous to HMPV Ala1313 (Figs. 2, 3, Supplementary Fig. 4). The RSV Cys1388 side chain sulfur mediates favorable interactions with the fluorophenyl (closest distance to phenyl 4.05 Å) of MRK-1. The contact is missing in HMPV with substitution to Ala. Instead, an empty space is present between the alanine and the inhibitor (closest distance to phenyl 4.82 Å), which could be filled with a water molecule that would be in an unfavorable hydrophobic environment. This sequence difference may also account for the different orientations observed for the fluorophenyl ring in the bound structures. Thus, the cryo-EM structures of the MRK-1 liganded RSV and HMPV polymerases provide insight into how the compound has broad spectrum inhibition of RSV and HMPV, while also suggesting why it has lower potency against HMPV.

### MRK-1 inhibits an early step of RSV transcription and genome replication

**Fig. 4:**
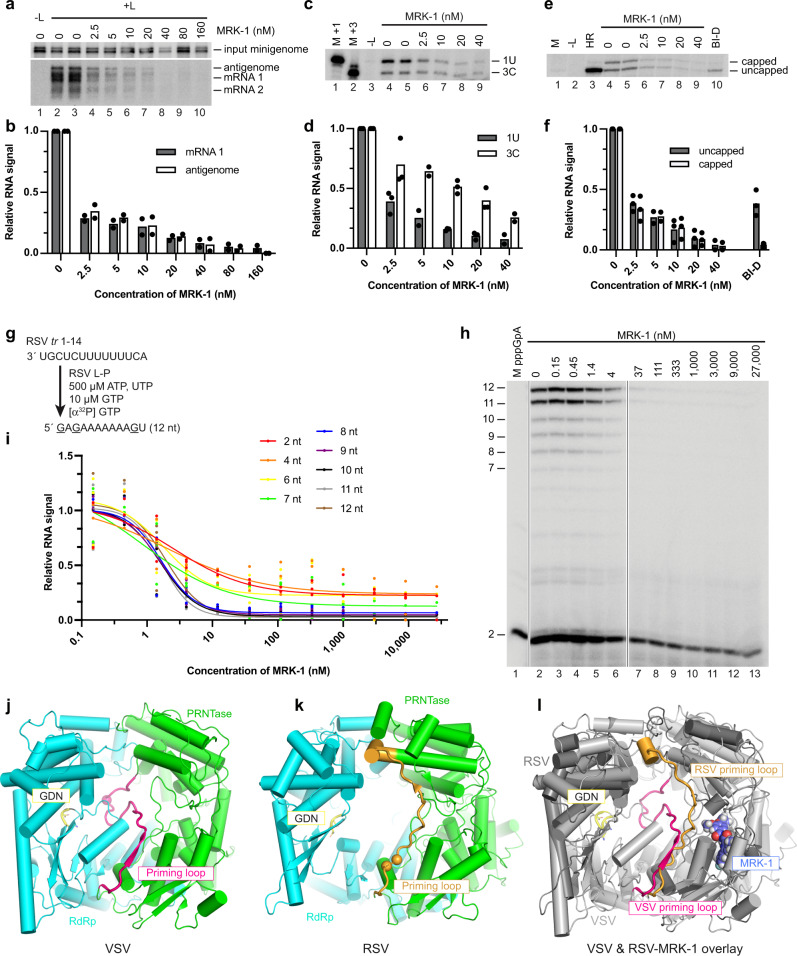
MRK-1 inhibits RSV synthesis initiation and early elongation. **a** Representative Northern blot analysis of minigenome-specific RNAs generated in the minigenome system. The blots show input minigenome, antigenome, and mRNAs 1 and 2 generated in the presence of varying concentrations of MRK-1. Lane 1 is a negative control in which the plasmid expressing the L protein was omitted from the transfection. **b** Quantification of mRNA 1 and antigenome RNA signal from two biological replicate Northern blot experiments, normalized to input minigenome. **c** Representative primer extension analysis of RNA generated from the minigenome in the presence of varying concentrations of MRK-1. The RNA was analyzed with a primer that corresponds to *leader* nucleotides 15–39 and detects RNA initiated at *leader* promoter positions 1U and 3C. Lanes 1 and 2 are 39 and 37 nt markers representing sizes of cDNA products generated from RNAs initiated at 1U or 3C, respectively. **d** Quantification of 1U and 3C products from two or three biological replicate experiments, normalized to RNA with no inhibitor. **e** Representative primer extension analysis of RNA generated from the minigenome analyzed with a primer that corresponds to nucleotides 12–31 of gene 1 and detects RNA initiated at the first *gene start* signal. The gel shows capped and uncapped RNAs generated in the presence of varying concentrations of MRK-1 or in the presence of 800 nM BI-D. Lane 1 shows a 31 nt marker representing the cDNA generated from uncapped RNA. Lane 2 shows RNA generated in transfections with an L protein containing H1338A and R1339A substitutions. **f** Quantification of capped and uncapped mRNAs from two or three biological replicate experiments, normalized to RNA generated by wt L protein with no inhibitor. **g** Schematic diagram illustrating the design of the RNA synthesis assay. **h** RNA synthesis products generated by polymerase in an in vitro RNA synthesis assay in the presence of varying concentrations of MRK-1, analyzed by denaturing polyacrylamide gel electrophoresis. A phosphorimage representing one of three independent experiments is shown. Vertical lines indicate where lanes were excised due to loss of the RNA pellet during purification. **i** Quantification of the different RNA products generated in vitro. Each value was normalized to minus inhibitor control, which was set to 1. The data show the mean individual data points of two or three independent experiments. **j**–**l** Comparisons of the VSV and RSV L structures, showing the *apo* structure of VSV L (PDB ID: 6U1X) (**j**), RSV L (PDB ID: 6PZK) (**k**), and an overlay of the RSV L_trunc_-MRK-1 and VSV L structures (PDB IDs 8FPI and 6U1X, respectively; **l**). Likely priming residues in VSV and RSV L proteins are indicated with spheres. In each case, the GDN motif in the RdRp domain is shown as yellow sticks. The indicated priming loops included the following amino acid residues: VSV L: 1146–1172 with Trp1167 indicated as the priming residue; RSV L: 1256–1282 with Pro1261 and Trp1262 indicated as the priming residues.

As noted above, the pneumovirus polymerase performs multiple steps to transcribe and replicate the viral genome. To transcribe the genome, the polymerase binds to a 3′ *leader* promoter region, initiates RNA synthesis at position 3C, and synthesizes and releases a short RNA^37^. The polymerase then scans the template to locate a *gene start* signal sequence at the beginning of the first gene, reinitiates RNA synthesis, and co-synthetically caps and methylates the RNA^35,37^. The polymerase can then elongate to a *gene end* signal sequence, polyadenylate, and release the RNA and continue to transcribe downstream genes by recognizing and responding to their *gene start* and *gene end* signals^13^. To replicate the genome, the polymerase binds to the *leader* promoter region and initiates RNA synthesis at position 1U. In this case, the polymerase generates encapsidated antigenome RNA, which in turn acts as a template for the synthesis of genome RNA^13^. To determine the mechanism of inhibition by MRK-1, it was analyzed using a replication-deficient RSV minigenome system. This assay allows transcription and replication to be measured as independent events, as described previously^38^ (Supplementary Fig. 5a). Northern blot analysis of minigenome-generated RNAs revealed that an increase in MRK-1 concentration resulted in a decrease in both mRNA and antigenome accumulation, suggesting that the ligand inhibits an activity common to both processes (Fig. 4a, b). Primer extension analysis was performed to examine the initiation and early elongation steps of RNA synthesis (i.e., steps that would lead to synthesis of an RNA long enough to bind to the primer). Analysis using a *leader*-specific primer that corresponds in sequence to *leader* nucleotides 15–39 showed that either initiation and/or early elongation from both 1U and 3C sites in the promoter was inhibited by MRK-1, although inhibition of RNA synthesis from 1U was slightly more pronounced (Fig. 4c, d). Analysis of RNA initiated from the *gene start* signal using a primer that binds to nucleotides 12–31 of the encoded mRNA also showed inhibition of pre-mRNA synthesis (Fig. 4e, f), with more pronounced inhibition than from the 3C transcription initiation site in the promoter (compare Fig. 4d and f), indicating that MRK-1 caused an additional inhibitory effect between early elongation from 3C and early elongation from the *gene start* signal. The primer extension method also allowed detection of the mRNA 5′ cap^35^ (Fig. 4e, f). Increasing the concentration of MRK-1 caused a decrease in levels of both uncapped and capped pre-mRNA, consistent with MRK-1 inhibiting either initiation and/ or early elongation but did not affect the ratio of uncapped to capped mRNAs. This contrasts with the alteration in the uncapped to capped RNA ratio if an L mutant containing a substitution in the PRNTase HR catalytic motif is used or if cells expressing WT L protein are incubated with compound BI-D, a small molecule RNA synthesis and capping inhibitor (Fig. 4e, lanes 3 and 10, f)^35,36^. In addition, Northern blot analysis showed that although reduced levels of RNA were generated in the presence of MRK-1, the RNAs that were generated were full-length (Fig. 4a). This is distinct from the smear of small abortive RNA products that are generated when cap addition is inhibited (Supplementary Fig. 5b)^17,35^, or the longer truncated RNAs that are observed if the RSV transcription elongation factor, M2-1, is omitted from a transfection reaction^39,40^. Thus, the minigenome studies showed that MRK-1 inhibited either initiation and/or early elongation of transcription and replication. In contrast, MRK-1 had no discernible effects on capping or late elongation. However, the caveat exists that the kinetics of MRK-1 and polymerase association might be too slow for MRK-1 to exert a detectable effect on the capping and late elongation activities of polymerase molecules that escaped inhibition during initiation and/or early elongation.

### MRK-1 inhibits both RSV RNA synthesis initiation and early elongation

To distinguish between an effect on initiation versus early elongation, the compound was tested in an in vitro assay designed to examine effects on RNA synthesis from position 3C of the promoter^41^. Reactions were performed by preincubating full-length RSV L-P complexes with different concentrations of MRK-1 and then initiating reactions by adding an RNA oligonucleotide representing nucleotides 1–14 of the RSV *trailer* promoter, and ATP, UTP, and GTP, with radiolabeled GTP included as a tracer (Fig. 4g). Following the reaction, RNA synthesis products were resolved to single nucleotide resolution by denaturing gel electrophoresis. Increasing the concentration of MRK-1 resulted in a decrease in the levels of the 2 nt pppGpA initiation product. However, although 50% initiation inhibition was observed with as low as ~4 nM MRK-1, some polymerase was refractory to initiation inhibition, even at a concentration of 27 µM (Fig. 4h, i). A similar initiation defect was observed in reactions with RSV L_trunc_-P (Supplementary Fig. 6). MRK-1 also elicited a defect in early elongation, as determined by examining products >2 nt in length. At lower concentrations of inhibitor (≤4 nM), initiation and elongation were similarly inhibited, but at higher concentrations, elongation was inhibited to a greater extent than initiation, with a more pronounced effect on products ≥7 nt in length (Fig. 4i). Thus, MRK-1 has two separable effects, inhibiting initiation and early elongation.

## Discussion

As noted above, the polymerases of different nsNSVs have been solved in different conformations^14–16,19–25^. It is well recognized that negative-strand RNA virus polymerases undergo a series of structural transitions corresponding to different stages of transcription and replication^28,42^. While the structural differences between different nsNSV polymerases could be due to virus-specific features, it is thought that the distinct domain and motif arrangements are due, at least in part, to the polymerases adopting structures representing different stages of RNA synthesis^26,28^. This hypothesis is supported by comparison of the structures of the EBOV polymerase in *apo* form and in association with a template-primer duplex^25^. A key difference between solved nsNSV polymerase structures is the priming loop position. Polymerase priming loops function during RNA synthesis initiation^28^. In the rhabdovirus *apo* polymerase structures, the priming loop projects into the RdRp active site, suggesting that in these cryo-EM structures, the polymerase is in a conformation poised for RNA synthesis initiation^21–23,43^ (Fig. 4j). In the pneumovirus *apo* polymerase structure a loop corresponding to the rhabdovirus priming loop is retracted away from the RdRp active site and integrated into the capping domain^14–16^ (Fig. 4k), closely resembling the putative priming loop conformation in the EBOV polymerase template-primer complex^25^, consistent with the pneumovirus polymerase being in an elongation conformation. A second loop, referred to as an “intrusion” loop, which contains the PRNTase HR catalytic motif^19^, is also positioned differently between the different polymerase structures (Supplementary Fig. 7a–d) and might adopt different conformations as the polymerase progresses through different stages of RNA synthesis^20^. Based on comparisons of nsNSV structures and the findings from this study, we suggest a model for inhibition by MRK-1. The cryo-EM structure shows MRK-1 occupying a pocket adjacent to the putative priming and intrusion loops (Fig. 4l, Supplementary Fig. 7e) and we propose that it elicits its effect by preventing necessary conformational changes in these loops. As noted above, the in vitro RNA synthesis assay revealed that MRK-1 has two separable effects on RNA synthesis: inhibition of (i) initiation (i.e., formation of the pppGpA dinucleotide) and (ii) elongation. It is thought that the pneumovirus *apo* polymerase structure represents the polymerase in a non-initiation conformation and we propose that MRK-1 inhibited RNA synthesis initiation by preventing the conformational changes that are necessary to position the priming loop in the RdRp active site and to enable rearrangement of the intrusion loop. According to this model, polymerase that was refractory to initiation inhibition was in a different conformation than that of the *apo* polymerase, perhaps with the priming loop already appropriately positioned so that the polymerase could engage in initiation. In this case, the polymerase could initiate RNA synthesis, but as it elongated and transitioned into the open conformation, MRK-1 binding prevented further early elongation, particularly as the polymerase elongated the RNA from 4 to 7 nucleotides. Thus, this model explains how MRK-1 functions as an inhibitor of both initiation and elongation.

MRK-1 binds adjacent to the catalytic RSV L His1338 in the PRNTase domain and causes re-orientation of the Arg1339 side chain that is required for capping activity, but it did not have a detectable effect on RSV polymerase cap addition or late elongation, albeit with the caveat described above. In addition, partial resistance was conferred by mutation of RSV L Ile1381 (Supplementary Table 1), which is also a resistance site for the BI-D capping inhibitor^36^. However, BI-D did not compete with MRK-1 (Fig. 1e, Supplementary Fig. 1b, d) and attempts to determine the co-structure of BI-D bound RSV L-P complexes by single particle cryo-EM failed to reveal any interpretable density for the compound. The PIV-5 polymerase adopts a different conformation from that of RSV, and based on the proximity of the HR motif to the transcript exit channel it has been suggested that the PIV-5 polymerase represents the structure that is poised for cap addition^19,26^. Based on these different strands of information, we speculate that the polymerase conformation required for capping activity is distinct from the *apo*-L-P conformation, and that the polymerase in the capping conformation does not bind MRK-1. Further structure studies of polymerase-RNA ligand complexes captured at different stages of transcription will be necessary to confirm this hypothesis.

In summary, the findings presented here provide valuable information regarding the interaction of a small molecule inhibitor that targets the polymerases of two genera of pneumoviruses, providing insight into the structure-function properties of the pneumovirus polymerases.

## Methods

### Determination of MRK-1 potency against clinical isolates of RSV by cytopathic effect assay

The antiviral potency of MRK-1 against the RSV A Ontario (ON1) 121301009 (A-BCM-10) and RSV B Buenos Aires (BA) 79362 (B-BCM-12) clinical strains (Baylor College of Medicine) was determined in a cytopathic effect assay. MRK-1 was placed in a 96-well microplate in a 11-point serial 3-fold dilution (in DMSO) and mixed with 5 × 10^3^ HEp-2 cells that had been infected at an MOI of 0.3 PFU/cell and incubated in DMEM with 10% heat-inactivated fetal bovine serum (FBS) at 37 ˚C, 5% CO_2_. At 7 days p.i. 100 µL of CellTiter-Glo (Promega) cell viability reagent was added, and the plates were subsequently incubated for 10 min at room temperature. The luminescence signal was measured with a Perkin Elmer Ensight multimode plate reader with a 1 s measurement time. EC_50_ values were determined by four parameter curve fitting using GraphPad Prism 8.

### RSV A and RSV B plaque reduction assays

Compound plates were prepared by dispensing 2 µL of test compound dissolved in DMSO into wells of a 96-well black tissue culture treated plate (Corning). Each compound was tested in a 10-point serial 3-fold dilution. Wells with DMSO (final concentration of 0.8%) or a compound at a concentration at which viral replication was completely inhibited were used as assay Min_E and Max_E control, respectively. HEp-2 cells (ATCC) were infected in cell suspension at an MOI of 0.001, distributed into the wells, and incubated at 37 ˚C, 5% CO_2_ for 1 h. Plates were then centrifuged at 200 × *g* for 10 min and cells were overlaid with a 125 µL/well of 1% methylcellulose solution in MEM containing 2% FBS and 100 U/mL penicillin-streptomycin. Plates were incubated at 37 ˚C, 5% CO_2_ for 3 days. Following incubation, the overlay was removed and cells were fixed with acetone. Plaques were detected by incubating cells with monoclonal antibodies 143-F3-1B8 RSVαF (Sino Biological) and 34C9 RSVαN (Sino Biological), followed by incubation with goat anti-mouse Alexa 488 antibody (Invitrogen). The plates were read on a Perkin Elmer Ensight multimode plate reader using brightfield and Alexa 488 settings with data analysis using a custom plaque algorithm. Antiviral EC_50_ values were determined using a 4-parameter logistic fit based on the Levenberg-Marquardt algorithm. Model 205, 4-Parameter Logistic.

### Generation of RSV A2-EGFP viruses

The RSV A2-EGFP reporter virus sequence is derived from the ATCC RSV A2 strain VR-1540. The reporter virus cDNA is placed under a T7 promoter. EGFP flanked by *gene start* and *gene end* sequences was inserted between the *P* and *M* genes. Four fragments spanning the T7 promoter, full-length RSV with EGFP, polyA/ribozyme/T7 terminator, with restriction enzyme sites were each synthesized and ligated with pSMART BAC vector (Lucigen). 0.5 µL of the ligation reaction mixture were transformed into BAC-optimized replicator v2.0 electrocompetent cells (Lucigen) using Gene Pulser (Bio-Rad) with the condition of 1.5 kV, 200 Ω, and 25 μF. pSMART RSV A2-EGFP positive clones were selected by colony PCR. The L I1381T mutation was introduced into the pSmart RSV A2-EGFP using Gibson assembly technique. The BAC of RSV A2 GFP virus, and the substitutions introduced to create the I1381T mutant, were confirmed by sequencing. Recovery of recombinant viruses was accomplished by mixing pSMART RSV A2-GFP or pSMART RSV A2-GFP L I1381T BAC DNA with pcDNA3.1 M2-1, N, P, and L at a ratio of 2:2:2:2:1. The DNA mixtures were transfected into BSR-T7 cells using TranIT-LT1 (Mirus) according to manufacturer’s instruction. After two weeks, the recombinant viruses were harvested and aliquoted.

### RSV A2-GFP and HMPV-GFP viral replication assays

GFP-expressing recombinant HMPV was generated in Dr. Buchholz’s lab^44^. Compound plates (384 well) were prepared by dispensing (202.5 nL/well) of test compounds solubilized in DMSO into flat clear bottom optical imaging microplates using an ECHO acoustic dispenser. Each compound was tested in a 10-point serial 3-fold dilution. Wells with DMSO (final concentration of 0.4%) or a test compound at a concentration at which viral replication was completely inhibited were used as viral replication assay Min_E and Max_E control, respectively. Calu-1 cells (ATCC) in the case of RSV, or Vero cells (ATCC) in the case of HMPV, were infected in suspension with RSV A2-GFP or HMPV-GFP virus at an MOI of 1.2 PFU/cell. 10 µL/well of 100% DMSO was dispensed to CellTiter-Glo (CTG) assay Max-E to controls wells and 50 µL/well prepared cells were dispensed into compound plates. Plates were covered with MicroClime lids loaded with 7.5 mL of assay media to minimize evaporation. Plates were lightly shaken for 10 min at room temperature, followed by incubation at 37 ˚C for 48 h. GFP-expressing cells were counted with an Acumen imaging system and a same-well CTG assay was performed by adding 10 µL/well of reconstituted CellTiter-Glo reagent (Promega G7573) and plates were read on PerkinElmer Envision. ActivityBase (IDBS) was used to analyze the raw data and antiviral EC_50_ and cytotoxicity CC_50_ values were determined using a 4-parameter logistic fit based on the Levenberg-Marquardt algorithm Model 205, 4-Parameter Logistic. The HEp-2, Calu-1, and Vero cells used for virology studies tested negative for mycoplasma using a MycoAlert Plus kit (Lonza).

### Expression of RSV and HMPV polymerases

The *L* and *P* genes from RSV and HMPV were codon optimized for insect cell expression and synthesized (GenScript). A PreScission protease cleavable 3xFLAG tag was added N-terminal to the L protein and a TEV cleavable 6xHis-tag was added C-terminal to the P protein. Genes for both full-length RSV L protein (aa 1–2165) and a truncated RSV L protein (aa 1–1481) were generated along with a full-length RSV P protein gene (aa 1–241). The gene for full-length HMPV L protein (aa 1–2005) was also generated along with the full-length HMPV P protein gene (aa 1–294). Full-length or truncated L and full-length P genes were subcloned into a pFastBac Dual vector (Thermo Fisher). Recombinant baculoviruses were prepared using the Bac-to-Bac system (Thermo Fisher) and protein was expressed by infecting Sf9 cells (Gibco) with the relevant baculovirus.

### Purification of RSV and HMPV polymerases

For large-scale purification both RSV full-length and truncated and HMPV full-length constructs utilized the same method. The Sf9 cell pellets were resuspended in lysis buffer (50 mM Tris pH 8.0, 200 mM NaCl, 1 mM TCEP, 10% glycerol) and were lysed by sonication. The lysate was then centrifuged, and protein complex captured with a prepacked Anti-FLAG M2 agarose column (Sigma) that had been equilibrated in lysis buffer. The protein complex was eluted with 100 µg/mL 3xFLAG peptide (Sigma) and subsequently loaded onto a Heparin HP column (Cytiva). The protein was gradient eluted (200 mM NaCl to 1 M NaCl) from the Heparin column. Additional purification utilized size-exclusion chromatography (Superose 6, Cytiva) using a buffer of 25 mM HEPES pH 7.4, 300 mM NaCl, 6 mM MgSO_4_, 0.5 mM TCEP. Final protein preparations were concentrated to 2 mg/mL for RSV and HMPV full-length complexes and 1.2 mg/mL for the RSV truncated complexes. Protein preparations were flash-frozen and stored at −70 ˚C until use.

### In vitro RNA synthesis reactions

Reactions to detect RNA synthesis products generated from position 3 of the *tr* promoter were performed with the following conditions: L-P complexes containing 300 ng RSV L or RSV L_trunc_ were incubated in 25 µL reactions containing 50 mM Tris pH 7.5, 8 mM MgCl_2_, 5 mM DTT, 10% glycerol, 2 μM RNA oligonucleotide RSV *tr*1-14 (Dharmacon), 500 μM each of ATP and UTP, and 10 µM GTP with 5 μCi [α-^32^P] GTP (3000 Ci/mmol)^41^. To analyze dinucleotide formation and elongation products in the presence of MRK-1, DMSO or various concentrations of inhibitor diluted in DMSO were included in the reaction mixtures. Reaction mixtures containing buffer, L-P complexes and MRK-1/ DMSO were preincubated at 30 ˚C for 10 min after which time RNA, NTPs, and [α-^32^P] GTP were added to start the reactions. The samples were incubated at 30 ˚C for a total of 1 h, heat inactivated at 95 ˚C for 5 min, and cooled on ice for 2 min. The concentration of GTP in the samples was increased to a final concentration of 500 µM and sample volumes were adjusted to 50 µL with RNase-free water. RNA was extracted with phenol-chloroform, precipitated with ethanol, and resuspended in 1X stop buffer containing deionized formamide, 20 mM EDTA, bromophenol blue, and xylene cyanol. Purified RNAs were migrated on 25% polyacrylamide gels containing 7 M urea in Tris-taurine-EDTA buffer, alongside molecular weight ladders. Molecular weight ladder representing RNA products initiated at position 3 of the tr RNA template was generated using an end-labeled RNA (5′ AUCAAAAACUGUGAAAAAAAGAG 3′). Specifically, 10 µL of end-labeled RNA was combined with 0.6 µL of yeast transfer ribonucleic acid (tRNA) (9.4 mg/mL) (Sigma-Aldrich) and 20 µL of 1X alkaline hydrolysis buffer (50 mM sodium carbonate pH 9.2, 1 mM EDTA) on ice. Solutions were aliquoted into 6 tubes of 5 µL and heated to 95 ˚C. After 1.5 min, one tube was removed from heat every minute. Immediately after removing a tube from the heat, 5 µL of 2X STOP buffer was added and the samples were placed on ice. Typically, 3 fractions of ladder were pooled together after determining which would give an evenly distributed ladder once combined. The ladder for the pppGpA dinucleotide was prepared using T7 RNA polymerase^41^. DNA oligonucleotides 5′ TCTATAGTGAGTCCGTATTA and 5′ TAATACGACTCACTATA were annealed to each other by mixing 4 μM of each oligonucleotide in Tris-EDTA pH 8.0, incubating at 90 ˚C for 3 min, then cooling to room temperature. 40 μL T7 RNA polymerase transcription reactions were performed in 40 mM Tris-HCl (pH 8.0), 20 mM MgCl_2_, 2 mM spermidine, 10 mM DTT, 0.1 mg/mL bovine serum albumin, 2 mM ATP, 1 mM CTP, 1 mM GTP with 1 μCi [α-^32^P] GTP, 0.1 μM DNA annealed oligos and 1 unit/μL T7 RNA polymerase (New England Biolabs). Reactions were incubated at 37 ˚C for 5 h. Samples were treated with 1 μL DNase I (New England Biolabs) for 10 min at 37 ˚C. 1 μL 0.5 M EDTA was added to the reactions. Products were subjected to phenol chloroform extraction and ethanol precipitation prior to use as markers. Following electrophoresis, gels were dried onto 3MM paper using a vacuum drier at 80 ˚C and analyzed by phosphorimager analysis. Radiolabeled RNA signals were quantified using Licor Image Studio Lite; background signal was subtracted from each sample and the final signal was normalized to the DMSO control sample.

### Radioligand binding assay

Binding assays were performed against full-length RSV L-P complex (either WT or an N812A substitution in the RdRp GDN motif). The binding curve of MRK-1 with RSV L-P was determined by titrating unlabeled compound against ^3^H-labeled MRK-1 in a filter binding assay. Competitive binding of various RSV inhibitors with ^3^H-MRK-1 was also evaluated using the same filter binding assay. Test compounds were added at a concentration of 10 µM to 5 µg/mL RSV polymerase (L-P protein complex) in 50 mM Tris, pH 7.5, 8 mM MgCl_2_, 5 mM TCEP, 10% (v/v) glycerol, 1% DMSO. All binding reactions were incubated for 30 min at room temperature in triplicate in 96-well polypropylene plates (Thermo Fisher). Following the incubation 10 nM of tritiated MRK-1 was added and the reaction was incubated at room temperature for ~4 h. The reactions were filtered through a Millipore GF/C filter plate (MAFCN0B50) pre-wet with wash buffer (10 mM HEPES pH 7.5, 25 mM KCl, 0.5 mM MgCl_2_, 10% PEG-8000) using a MultiScreen vacuum manifold. The filter plate was subsequently washed 10 times under vacuum with 100 µL/well of wash buffer (1 mL total/well). After washing, the membrane of the filter plate was allowed to dry for approximately 30 min at 37 ˚C in a vacuum oven. The bottom of the plate was sealed with opaque sealing tape and 80 µL of Microscint-20 liquid scintillation fluid (Perkin Elmer) was added to each well of the plate. The top of the plate was then sealed with clear sealing tape and the plate was counted in the Top Count NXT HTS with a 1 min read time/well.

### Cryo-EM data collection

For grid preparation, a 1:1 mix of either RSV L_trunc_-P or HMPV L-P complex samples at ~1.2 mg/mL with a solution containing 25 mM Na-HEPES pH 7.4, 300 mM NaCl, 6 mM MgCl_2_, 0.5 mM TCEP, 2% v/v deuterated DMSO, and 2 mM MRK-1 were made and allowed to incubate for approximately 5–10 min. 3 µL of the mixture was deposited on QuantiFoil R1.2/1.3 300 Au grids which were previously glow discharged with an EasyGlow, set at default parameters, and blotted and flash-frozen in liquid ethane using a Vitribot Mk IV set at the manufacturer-recommended settings. All data were collected on a Krios microscope operating at 300 kV. For the RSV protein, a Falcon 3 detector was used. The pixel size was set to 1.06 Å. 40 frames were collected for each micrograph, with a total exposure dose of 34.5 e^−^/Å^2^. 1920 micrographs out of 2219 were used for particle picking. For HMPV, a Gatan K3 detector was used, the pixel size was 0.815 Å, 40 frames were measured for each micrograph, 4.981 micrographs out of 9577 were selected for processing, the dose was 45 e^−^/Å^2^. Motion correction, CTF estimation, “blob”-based or template-based picking, 2D classification, ab initio reconstruction, homogeneous 3D refinement, and non-uniform 3D refinement were performed in cryoSPARC v2 and v3 for RSV and HMPV polymerases, respectively. For RSV, 1,540,913 particles were picked of which 589,457 among the 13 best 2D classes were retained for ab initio reconstruction and subsequent calculations which resulted in a 2.39 Å GFSCS resolution. The numbers for HMPV were 5,087,965 particles picked, 524,654 retained from 10 2D classes, and a resolution of 2.74 Å.

### Analysis of RSV transcription and replication using a minigenome system

Minigenome RNA synthesis was reconstituted in BSR-T7/5 cells^45^, kindly provided by Dr. Karl-Klaus Conzelmann (Ludwig Maximilians University). The cells tested negative for mycoplasma using an EZ-PCR Mycoplasma Detection Kit (Biological Industries). The 970 nt minigenome used contains in 3′ to 5′ order: the *leader* promoter region, *NS1 gene start* signal, *NS1* non-translated region, *chloramphenicol acetyltransferase* gene containing an inserted RSV N-P gene junction to create two genes of 580 nt and 190 nt in length, *L gene end* signal, and trailer region, with a 2C-to-G substitution to limit the polymerase to a single round of RNA replication. The minigenome is under the control of a T7 promoter (separated from the trailer region by 3G residues) and flanked by a hepatitis delta virus ribozyme. The identities of the antigenome and mRNA products generated from the RSV minigenome template were validated in previous work by mutating the *leader* promoter region and altering the *gene start* and *gene end* signals^38,40,46^. Cells in 6-well plates were transfected with 200 ng/well minigenome plasmid, and pTM1 plasmids expressing RSV N (400 ng/well), P (200 ng/well), M2-1 (200 ng/well) and codon optimized L (100 ng/well) using Lipofectamine 3000 (Thermo Fisher), using the manufacturer’s instructions. Before addition of transfection mix to the cells, DMSO or various concentrations of inhibitors diluted in DMSO were added to the mix. Cells were incubated at 37 ˚C. After 18 h, the transfection mixture was replaced with Opti-MEM containing 2% FBS and either fresh DMSO or inhibitors at the appropriate concentration. At 48 h post-transfection, cells were harvested to isolate total intracellular RNA. RNA samples were prepared using either the Monarch Total RNA Miniprep Kit with on-column DNase treatment (NEB) or TRIzol (Invitrogen) according to the manufacturer’s instructions, except that in the Trizol purification procedure, the RNA was subjected to additional purification by acid phenol-chloroform extraction and ethanol precipitation. RNA from each sample was subjected to Northern blot analysis^35^. Between 0.75 and 2 µg of total RNA of sample (the amount of RNA was consistent between each sample in a biological replicate) was migrated on a two-tier 1.5% agarose formaldehyde gels in 1X MOPS buffer. The top and bottom tiers included the same positive control +L, DMSO only control RNA, and the bottom tier included a colored molecular weight ladder (Dynamarker prestain marker for RNA High, Diagnocine). Following electrophoresis, the RNAs were transferred onto 0.2 µm Nytran N nylon blotting membranes (Sigma-Aldrich) in a buffer of 3 M NaCl, 8 mM NaOH in a Whatman TurboBlotter downward capillary transfer system. Membranes were washed in saline sodium citrate buffer and subjected to UV-cross-linking. Blots were probed with [α-^32^P] incorporated riboprobes specific to the positive or negative sense minigenome RNA, as indicated. Northern blots were analyzed by phosphorimager analysis. The signal from -L samples were subtracted from each sample value, and this value was then normalized to input minigenome RNA, as determined Northern blotting, and to the mean of the DMSO control samples. The Northern blots were also exposed to autoradiographic film, and the molecular weight markers were marked onto the film by alignment to the colored ladder on the blot.

Primer extension reactions were performed on 2–3.8 µg of total intracellular RNA isolated from minigenome transfected cells (the amount of RNA was consistent between each sample in a biological replicate). RNAs initiated at positions 1U and 3C were detected using a ^32^P end-labeled primer with the sequence 5′ TTTGGTTTATGCAAGTTTGTTGTAC corresponding to nucleotides 15–39 of the leader region. These primer extension reactions were performed using 0.2 µM primer, 500 µM each dNTP, and Sensiscript reverse transcriptase (Qiagen), according to manufacturer’s instructions^41^. Products were migrated on a 6% polyacrylamide 7 M urea gel in Tris-borate-EDTA buffer alongside markers consisting of ^32^P end-labeled DNA oligonucleotides corresponding to the length and sequence of cDNA representing RNA initiated at position 1U or 3C (39-nt or 37-nt, respectively). RNA initiated at the *gene start* signal was detected using a ^32^P end-labeled primer with the sequence 5′ AAGTGGTACTTATCAAATTC, corresponding to nucleotides 12–31 of the first mRNA, using Moloney murine leukemia virus reverse transcriptase (Promega) according to the manufacturer’s instructions, except that each dNTP was included at a concentration of 5 µM. *Gene start* primer extension products were subjected to electrophoresis on 20% acrylamide gels containing 7 M urea gel in tris-borate-EDTA buffer, alongside a ^32^P end-labeled marker corresponding to the length and sequence of cDNA representing the first 31-nt of mRNA 1. Gels were dried onto 3MM paper using a vacuum drier at 80 ˚C Primer extension products were detected and quantified by phosphorimager analysis. RNA signal was quantified using Licor Image Studio Lite. The signal from -L samples were subtracted from each sample value and to the mean of the DMSO control samples.

### Figure preparation

Structure visualization and figure preparation were performed using PyMOL Molecular Graphics Sytem, Version 2.5.4 (Schrödinger LLC), UCSF Chimera, and APBS (Adaptive Poisson-Boltzmann Solver)^47–49^.

### Statistics and reproducibility

Graphs were prepared using GraphPad Prism version 9.5.1. No statistical analyses were performed on the data provided. All graphs show the mean as a bar or fitted-curve and individual data points in dot plot formation; refer to the legends for the number of biological and technical replicates performed. Biological replicates of virology experiments were involved infections performed on different days or at different times of the same day. Biological replicates of minigenome experiments involved transfections performed on different days with cells of a different passage number. Biological replicates of in vitro RNA synthesis assays were performed either on different days with separate master mixes or at different times on the same day with separate master mixes. The same RSV polymerase preparation was used for all in vitro RNA synthesis assays. The source data for all graphs presented is included as Supplementary Data 1.

### Reporting summary

Further information on research design is available in the Nature Portfolio Reporting Summary linked to this article.

## Supplementary information

Supplementary Information

Description of Additional Supplementary Files

Supplementary Data 1

Reporting Summary

## Data Availability

Structure coordinates are available from the RCSB Protein Data Bank (PDB) under accession codes 8FPI (RSV L_trunc_-P in complex with MRK-1) and 8FPJ (HMPV L-P in complex with MRK-1), and the electron density map from the Electron Microscopy Data Bank (EMDB) under accession codes EMD-29365 (RSV L_trunc_-P in complex with MRK-1) and EMD-29366 (HMPV L-P in complex with MRK-1). Unedited and uncropped gels and blots for Figs. 1 and 4, and Supplementary Figs. 5 and 6 are shown in Supplementary Figs. 8 to 11, respectively. Mass spectra of the MSD-RSV radiolabel tool and ^3^H-labeled MRK-1 tracer are shown in Supplementary Figs. 12 to 13, respectively. The source data for the graphs that are presented are in Supplementary Data 1. All other data are available from the corresponding author on reasonable request.
