# Supplementary material for: Conserved allosteric inhibitory site on the respiratory syncytial virus and human metapneumovirus RNA-dependent RNA polymerases
Source: Commun Biol. 2023 Jun 19;6:649. doi: 10.1038/s42003-023-04990-0 (PMC10279679; doi:10.1038/s42003-023-04990-0)
Supplement: Supplementary file 1 — Supplementary Information [file 42003_2023_4990_MOESM1_ESM.pdf]

Supplementary Information

Conserved Allosteric Inhibitory Site on the Respiratory Syncytial Virus and Human Metapneumovirus RNA-Dependent RNA Polymerases

Victoria A. Kleiner[1#], Thierry O. Fischmann[2#], John A. Howe[2], Douglas C. Beshore[2], Michael J. Eddins[2], Yan Hou[2], Todd Mayhood[2], Daniel Klein[2], Debbie D. Nahas[2], Bob J. Lucas[2], Xi, He[2], Edward Murray[2], Daphne Ma[2], Krista Getty[2], Rachel Fearns[1]*

[1] Department of Virology, Immunology & Microbiology, National Emerging Infectious Diseases Laboratories, Boston University Chobanian and Avedisian School of Medicine, Boston, MA, USA

[2] MRL, Merck & Co., Inc., Rahway, NJ, USA

[#] These authors contributed equally to the work.

*Corresponding author: rfearns@bu.edu

Supplementary Figures

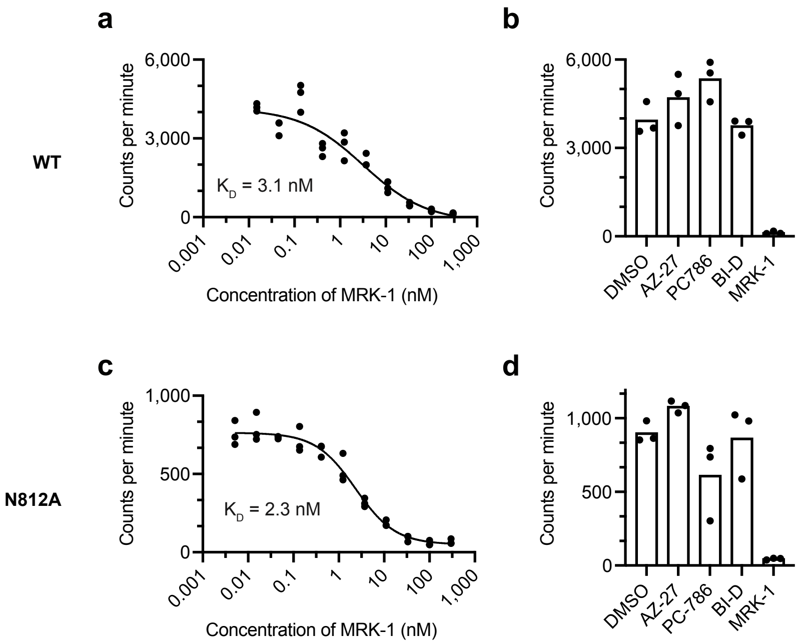

Supplementary Figure 1. Determination and comparison of MRK-1 binding affinity against other identified inhibitors. (a, c) Titration of unlabeled MRK-1 against radiolabeled MRK-1 to determine the affinity of binding to wild-type (WT) RSV L-P (a) or RSV L_{N812A}-P, in which the L protein has an amino acid substitution in the GDN motif of the RNA dependent RNA polymerase domain (c). The data show the mean and individual data points of three technical replicates. Dissociation constant (K_D) is denoted under each curve. (b, d) Analysis of competition between unlabeled NNIs and radiolabeled MRK-1 for binding to (b) WT RSV L-P or (d) RSV L_{N812A}-P. The data show the mean and individual data points of three technical replicates.

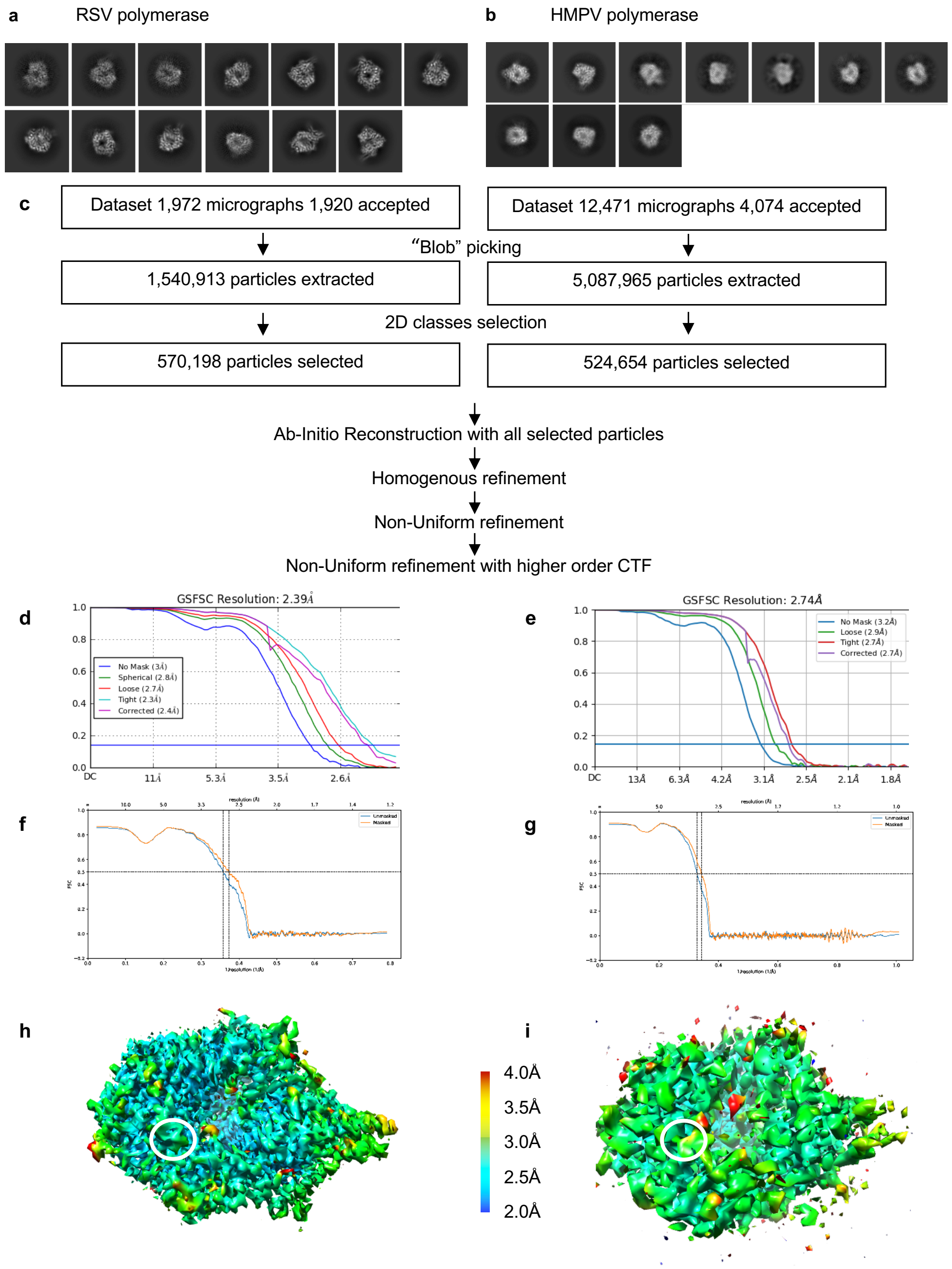

Supplementary Figure 2. Cryo-EM workflow and statistics. (a, b) Selected 2D classes of the RSV (a) and HMPV (b) polymerases. (c) Data processing workflow in CryoSPARC. (d) FSC curves for RSV polymerase: FSC graphs after the last non-uniform refinement step in CryoPSARC inferred from comparison of two half-map; and (f) FSC curves from comparison of model and maps, calculated after real space refinement with Phenix. (e) and (g) FSC curves for HMPV polymerase, as described for the RSV polymerase in d and f. (h) RSV polymerase density map colored by local resolution as estimated with CryoSPARC. The thresholds are

indicated: red, yellow, green, cyan, blue for 4.0Å, 3.5Å, 3.0Å, 2.5Å, and 2.0Å, respectively. (i) HMPV polymerase density map colored by local resolution, with same thresholds as (h).

a

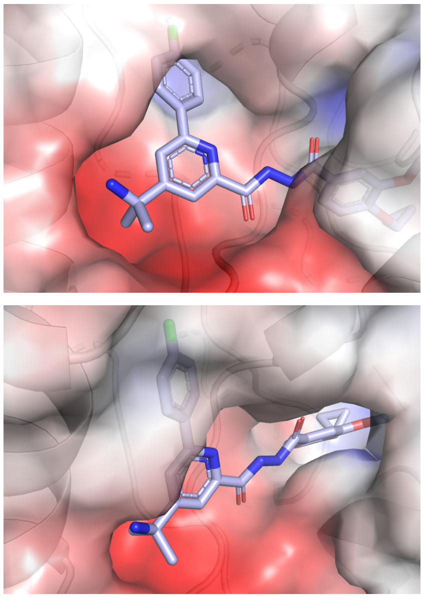

b

Supplementary Figure 3. MRK-1 binding pocket in the RSV polymerase. Surface representation of RSV L showing the binding pocket of MRK-1 in the PRNTase domain (PDB ID: 8FPI). Panels (a) and (b) show two different perspectives. The PRNTase domain is colored by electrostatic potential calculated with APBS.

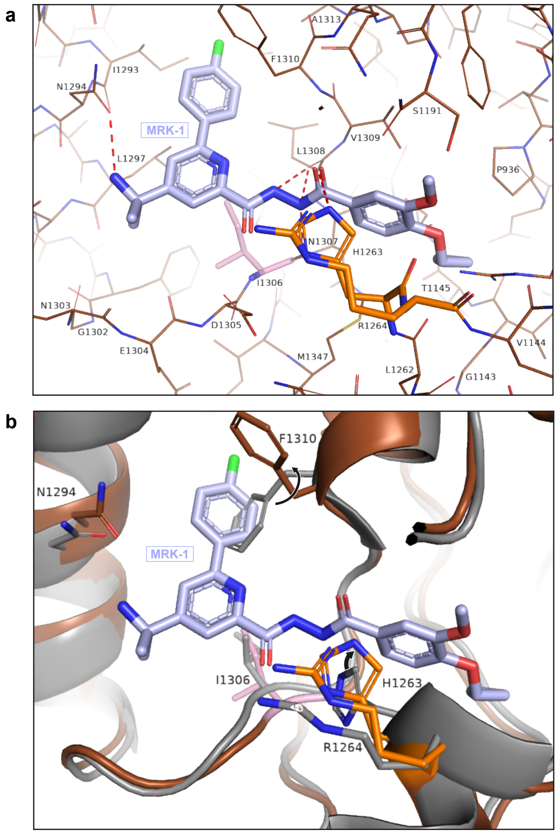

Supplementary Figure 4. Structure of HMPV L in complex with MRK-1. (a) Magnified view of the MRK-1 binding pocket within HMPV L (PDB ID: 8FPJ). Ile1306, which corresponds to RSV L Ile1381, is shown in pink and the HR motif in orange. (b) The MRK-1 liganded HMPV L structure superimposed on the *apo* L structure (PDB ID: 6U5O). The *apo* HMPV L structure is shown in grey, and the MRK-1 bound structure is shown in brown. Rearrangements are indicated with black arrows.

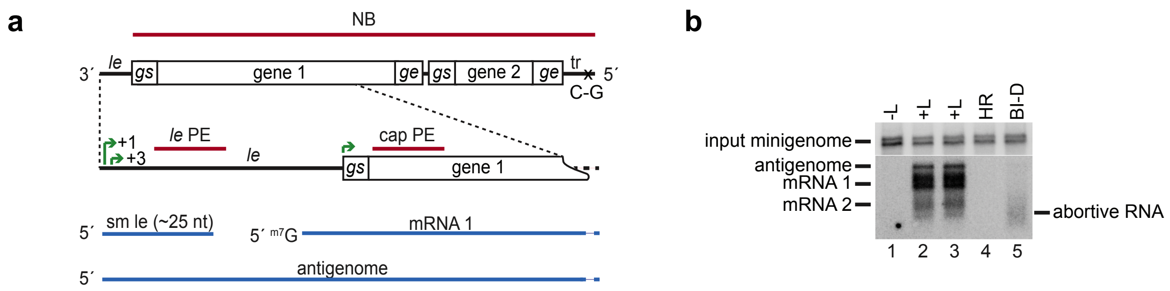

Supplementary Figure 5. Detection of abortive RNAs generated in the presence of BI-D. (a) Schematic diagram (not to scale) of the dicistronic, replication-deficient minigenome used to detect transcription and replication products generated by the RSV polymerase in the presence of MRK-1 or BI-D. The 970 nt minigenome contains the RSV *leader* (*le*) and trailer (tr) regions at the 3′ and 5′ ends, respectively, and the two genes of 580 nt and 190 nt, comprised of sequence from the bacterial *chloramphenicol acetyltransferase* gene, are each flanked with RSV *gene start* (*gs*) and *gene end* (*ge*) sequences. Sequences with a *cis*-acting function are italicized. This minigenome contains a C-to-G substitution at position 2 relative to the 5′ end of the tr region to prevent multi-cycle replication. The probes used for Northern blot (NB) analysis or primer extension (PE) analysis are shown in red, and replication and transcription products are shown in blue. The ~ 25 nt small leader RNA generated from position 3C of the *le* promoter is denoted sm le. (b) Representative Northern blot analysis of minigenome-specific RNAs generated in the absence or presence of 800 nM BI-D. BI-D causes production of abortive RNAs smaller than the RNAs generated in the absence of BI-D. Lane 4 shows RNA generated in transfections with a plasmid expressing L protein containing H1338A and R1339A substitutions. Lane 1 is a negative control in which the plasmid expressing L protein was omitted from the transfection.

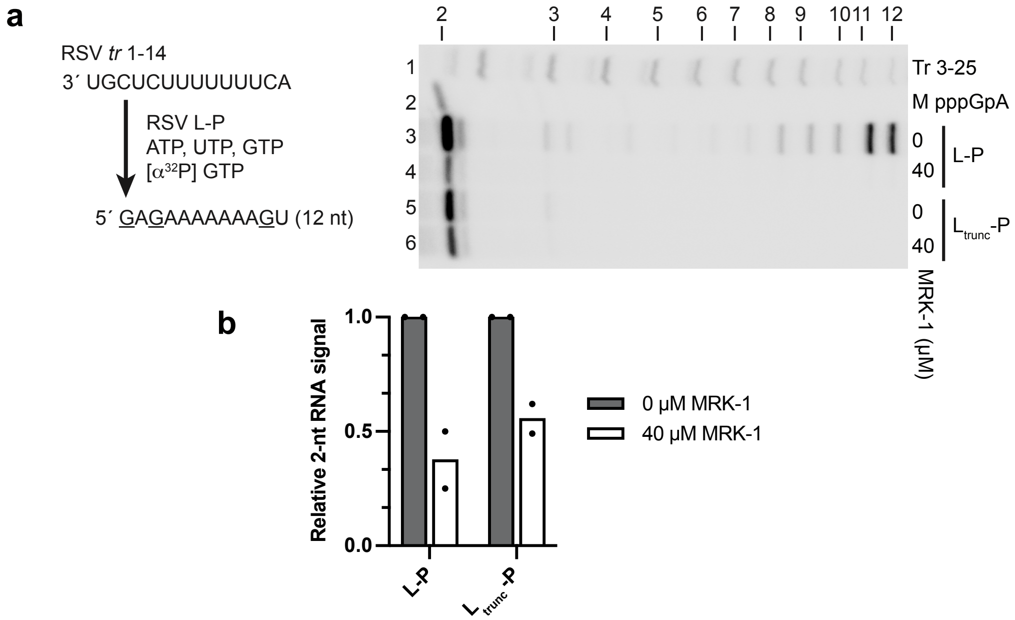

Supplementary Figure 6. MRK-1 inhibits RNA synthesis initiation and elongation by RSV L_{trunc}-P. (a) RNA synthesis activities of RSV L-P and L_{trunc}-P from position 3C of the RSV *trailer* promoter in the absence or presence of 40 μM MRK-1. A phosphorimage representing one of two independent experiments is shown. Lane 1 shows a ladder representing products generated from position 3C of a trailer 1-25 promoter. Note that these RNAs have a 5´ OH and products ≤ 7 nt in length migrate differently than RNA with a 5´ triphosphate. Lane 2 shows a marker for the pppGpA dinucleotide. (b) Quantification of the dinucleotide products generated *in vitro*. Each value was normalized to minus inhibitor control, which was set to 1. The data show the mean and range of two independent experiments.

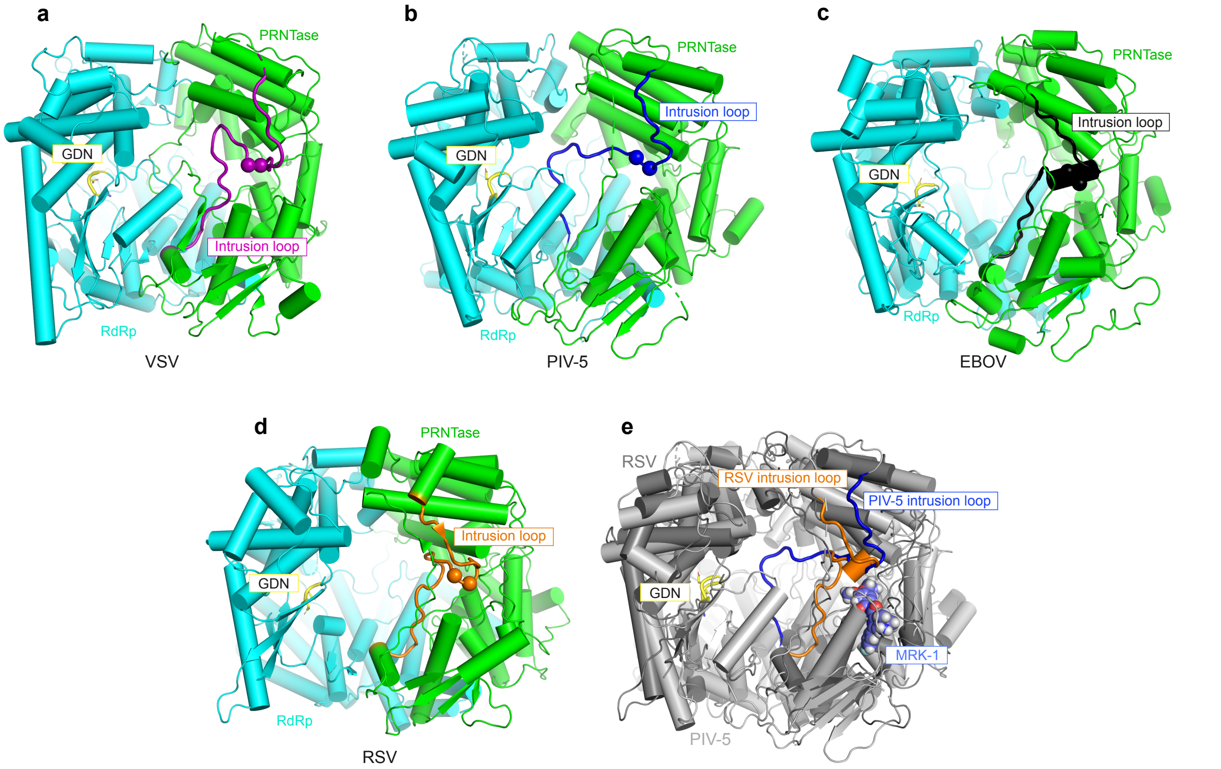

Supplementary Figure 7. Comparison of intrusion loop positioning in VSV, PIV-5, EBOV, and RSV polymerase structures. Comparisons of the VSV, PIV-5, EBOV, and RSV L structures, showing the *apo* structure of VSV L (PDB ID: 6U1X) (a), PIV-5 L (PDB ID: 6V85) (b), EBOV L (PDB ID: 7YES) (c), RSV L (PDB ID: 6PZK) (d), and an overlay of the PIV-5 L and RSV L_{trunc}-MRK-1 (PDB ID: 8FPI) structures (e). In each case, the GDN motif in the RdRp domain is shown as yellow sticks. The indicated intrusion loops included the following amino acid residues: VSV L: 1217-1243; PIV-5 L: 1282-1307; EBOV L: 1259-1285; RSV L: 1328-1354. The HR motif is shown as magenta spheres in VSV L (His1227, Arg1228), blue spheres in PIV-5 L (His1292, Arg1293), black spheres in EBOV L (His 1269, Arg 1270), and orange spheres in RSV L (His1338, Arg1339).

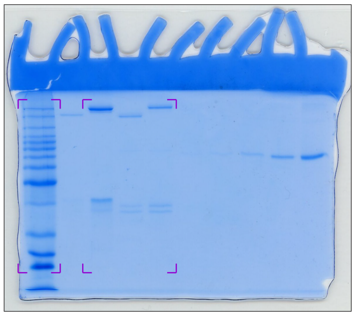
Figure 1c

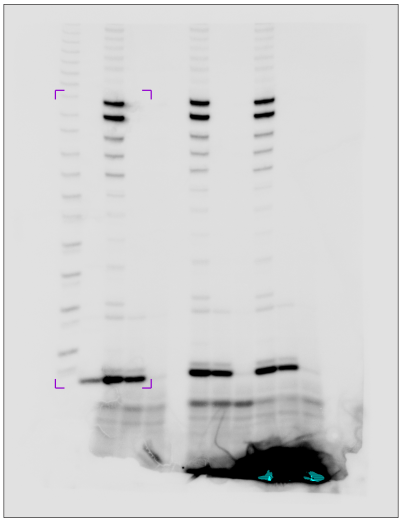
Figure 1g

Supplementary Figure 8. Original source images for data in Figure 1. The figure shows the uncropped and unedited images of gel electrophoresis data. The purple marks indicate how the images were cropped for the manuscript figure.

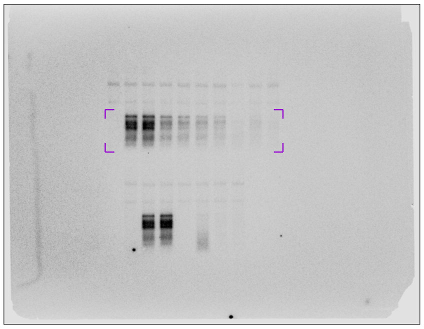

Figure 4a

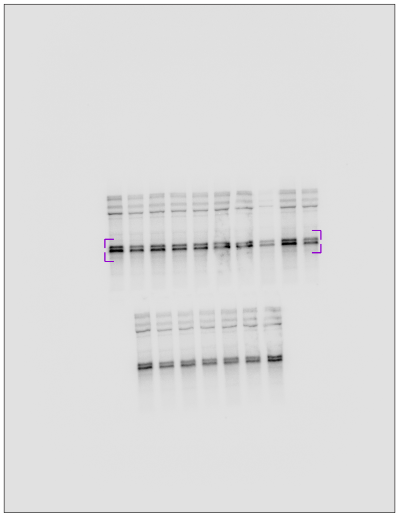

Figure 4a

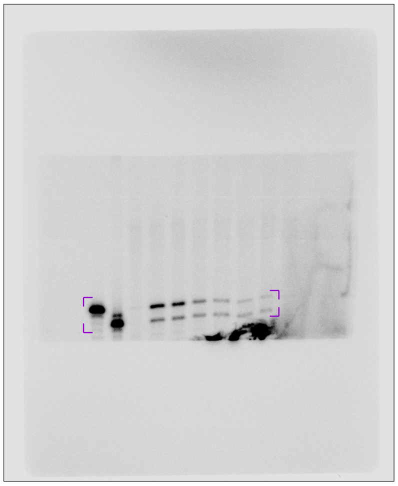

Figure 4c

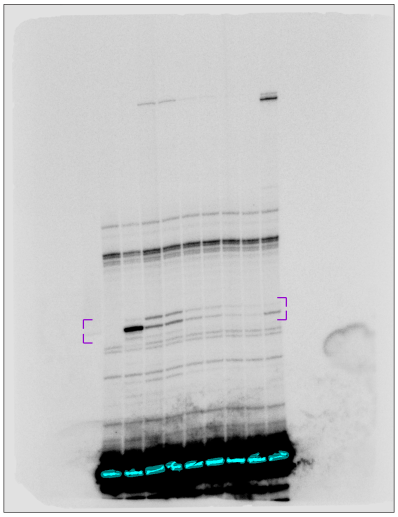

Figure 4e

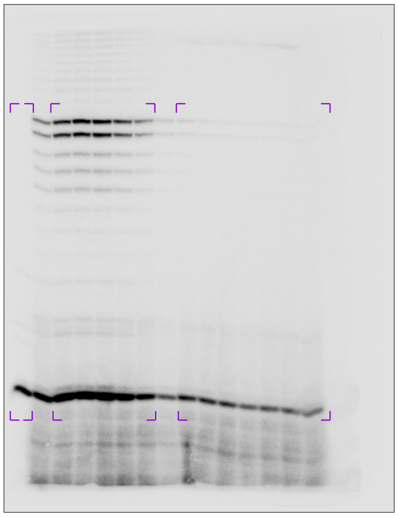

Figure 4g

Supplementary Figure 9. Original source images for data in Figure 4. The figure shows the uncropped and unedited images of gel electrophoresis data. The purple marks indicate how the images were cropped for the manuscript figure. See Supplementary Figure 10 for information regarding molecular weight ladders in Figure 4a.

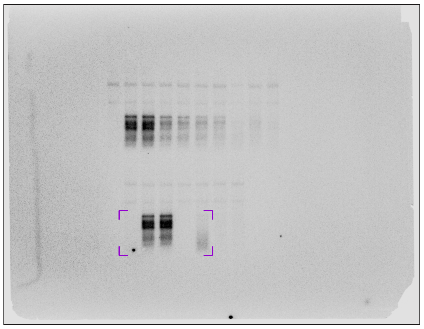

Supplementary Figure 5b phosphorimage

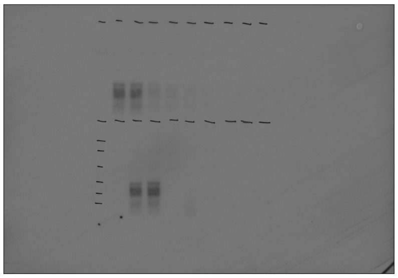

Supplementary Figure 5b autoradiography film

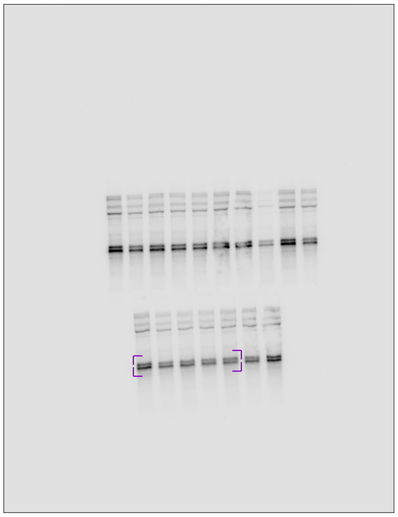

Supplementary Figure 5b phosphorimage

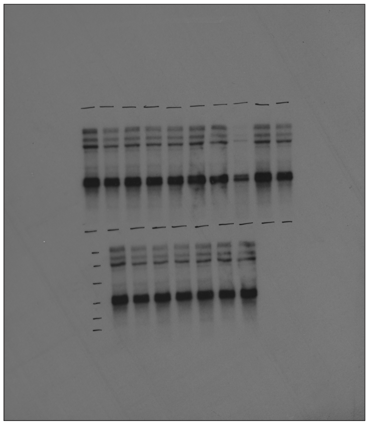

Supplementary Figure 5b autoradiography film

Supplementary Figure 10. Original source images for Supplementary Figure 5. The figure shows the uncropped and unedited images of electrophoretic gels exposed to autoradiography film and phosphorimage storage screens. The purple marks indicate how the images from a phosphorimage were cropped for the manuscript figure. The markings on the autoradiograms show the locations of the lanes and the ladder (marked by hand). The ladder was marked onto the autoradiograms by alignment with the colored ladder on the Northern blot membrane. From top to bottom the size markers are 8 kb, 4 kb, 2 kb, 1 kb, 0.5 kb, and 0.2 kb. Note that the same duplicate wt polymerase, DMSO control sample RNAs were used for the top and bottom tiers of each gel.

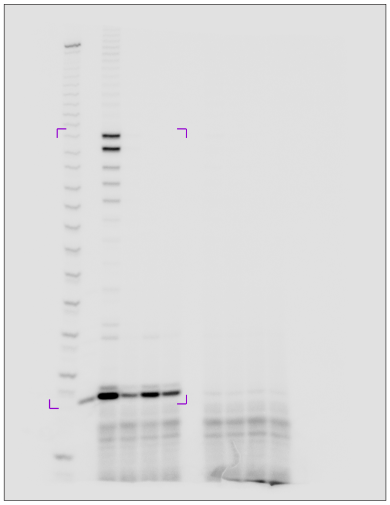

Supplementary Figure 6a

Supplementary Figure 11. Original source images for Supplementary Figure 6. The figure shows the uncropped and unedited images of gel electrophoresis data. The purple marks indicate how the images were cropped for the manuscript figure.

Supplementary Tables

Assay	Virus strain	EC_{50} / CC_{50} mean ± SD (nM)
RSV-EGFP expressing Calu-1 cells	RSV-A (Long A2)	2.1 ± 0.8 (N = 12, n = 1)
Plaque reduction assay HEp-2 cells	RSV-A (Long A2)	3.9 ± 1.4 (N = 4, n = 2, 4, 2, 4)
	RSV-B (Washington/18537/62)	3.3 ± 0.9 (N = 3, n = 4, 2, 4)
Cytopathic effect in RSV-infected HEp-2 cells	RSV-A 121301009 (ON1) B-BCM-10	4.3 ± 1.4 (N = 2, n = 3)
	RSV-B 79362 (BA) B-BCM-12	1.3 ± 0.6 (N = 2, n = 3)
RSV-EGFP L/I1381T Calu-1 cells	RSV-A (Long A2)	372 ± 29 (N = 3, n = 3, 5, 5)
HMPV-EGFP expressing Vero cells	HMPV-GFP	185 ± 23 (N = 3, n = 5)
CTG cytotoxicity assay Calu-1 cells	—	16,659 ± 7,097 (N = 12, n = 1)
CTG cytotoxicity assay Vero cells	—	15,373 ± 1,314 (N = 12, n = 1)

EC_{50} = 50% of maximal effective concentration; CC_{50} = 50% of maximal cytotoxic concentration; N = biological replicates; n = technical replicates
EGFP = enhanced green fluorescence protein; CTG = CellTiter-Glo

Supplementary Table 1. MRK-1 potency against different viruses as determined in different cell lines and assays.

	apo RSV L-P (PDB 6PZK)	RSV L_{trunc}-P-MRK-1	*apo* HMPV L-P (PDB 6U5O)	HMPV L-P-MRK-1
apo RSV L-P	0	0.469 Å	1.138 Å	1.166 Å
RSV L_{trunc}-P-MRK-1	0.738 Å	0	1.201 Å	1.145 Å
apo HMPV L-P	0.949 Å	1.018 Å	0	0.735 Å
HMPV L-P-MRK-1	0.781 Å	0.720 Å	0.746 Å	0

Supplementary Table 2. R.M.S.D. between Cα positions. The numbers below the diagonal from top left to bottom right corners are the results of superpositions of residues in the immediate vicinity of the ligand, the number above are for the entire PRNTase domain. Numbers calculated with PyMOL using a 3 Å distance cut-off.

Supplementary Note 1

Compound Preparation

All reactions were performed under nitrogen atmosphere in round bottomed flasks. Proton NMR spectra were recorded on Varian Unity 400 or VRX-400 spectrometers (400 MHz), a Varian Unity or Varian Plus (500 MHz) spectrometer, or a Bruker AVANCE III HD 700 (700 MHz) spectrometer. Chemical shifts are reported in parts per million (δ) downfield from tetramethylsilane as an internal standard. Samples were dissolved in acetonitrile:water or used as provided, and ionized by use of electrospray ionization (ESI) yielding [M+H]. External calibration was accomplished with oligomers of polypropylene glycol (PPG, average molecular weight 425 Da). Column chromatography was performed on ISCO Combiflash purification systems employing Silicycle prepacked silica gel cartridges and analytical thin layer chromatography was performed on EM Science Kieselgel 60 F254 plates. Solvents and reagents were obtained from commercial sources and used without further purification. The reported yields are the actual isolated yields of purified material and are not optimized.

Preparation of MRK-1

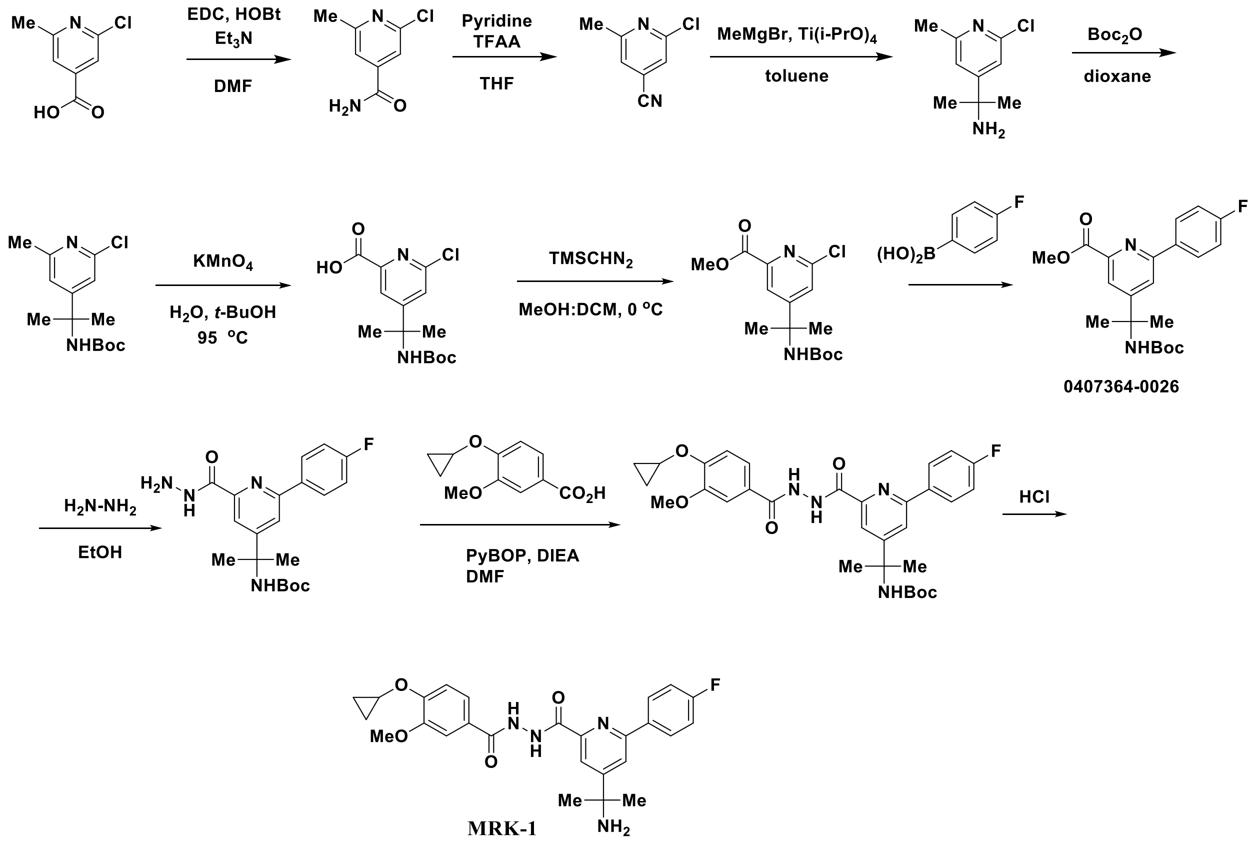

Preparation of 2-chloro-6-methylpyridine-4-carboxamide: Into a 20-L 4-necked round-bottom flask purged and maintained with an inert atmosphere of nitrogen, was placed a solution of 2-chloro-6-methylpyridine-4-carboxylic acid (350 g, 2.04 mol) in *N,N*-dimethylformamide (7 L). This was followed by the addition of amine hydrochloride (327 g, 6.12 mol, 3.00 equiv) at 0 °C, followed by triethylamine (619 g, 6.12 mol, 3.00 equiv), (3-dimethylamino-propyl)-ethyl-carbodiimide hydrochloride (469 g, 2.45 mol, 1.20 equiv), 1-hydroxybenzotriazole (330 g, 2.45 mol, 1.20 equiv). The resulting solution was stirred for 18 hours at room temperature. The mixture was treated with aqueous saturated sodium bicarbonate (7 L) and extracted with ethyl acetate (3 x 7 L). The combined organic extracts were dried with magnesium sulfate, filtered, and concentrated *in vacuo*, providing the titled compound (190.2 g, 55%) as a brown solid.

Preparation of 2-chloro-6-methylpyridine-4-carbonitrile: Into a 20-L 4-necked round-bottom flask purged and maintained with an inert atmosphere of nitrogen, was placed a solution of 2-

chloro-6-methylpyridine-4-carboxamide (190 g, 1.11 mol) in tetrahydrofuran (3.8 L). This was followed by the addition of triethylamine (338 g, 3.34 mol, 3.00 equiv) dropwise with stirring at 0 °C. To this was added a solution of trifluoroacetic acid anhydride (351 g, 1.67 mol, 1.50 equiv) in tetrahydrofuran (700 mL) dropwise with stirring over 30 minutes. The resulting solution was warmed to ambient temperature and stirred for 1 hour. The mixture was treated with aqueous saturated sodium bicarbonate (3.8 L) and extracted with ethyl acetate (3 x 4 L). The combined organic extracts were washed once with aqueous saturated sodium chloride (4 L), dried with magnesium sulfate, filtered, and concentrated *in vacuo*. The residue was subjected to silica gel chromatography (eluting with ethyl acetate/petroleum ether; 1:10), providing the titled compound (151 g, 89 % yield) as a white solid.

Preparation of *tert*-butyl *N*-[2-(2-chloro-6-methylpyridin-4-yl)propan-2-yl]carbamate: Into a 10-L 4-necked round-bottom flask purged and maintained with an inert atmosphere of nitrogen, was placed a solution of 2-chloro-6-methylpyridine-4-carbonitrile (150 g, 983 mmol) in toluene (3 L). The mixture was cooled to 0 °C, treated with methyl magnesium bromide (1.31 L, 4.0 equiv) dropwise. The mixture was warmed to ambient temperature and stirred for an additional 30 minutes. The mixture was cooled to 0 °C, treated with $Ti(O^iPr)_4$ (279 g, 983 mmol, 1.00 equiv), warmed to ambient temperature and stirred for 18 hours. The mixture was treated with aqueous saturated ammonium chloride (3 L) and extracted with ethyl acetate (3 x 3 L). The combined organic extracts were washed once with brine (3 L), dried with magnesium sulfate, filtered, and concentrated *in vacuo*, providing the titled compound as a yellow oil.

Preparation of *tert*-butyl *N*-[2-(2-chloro-6-methylpyridin-4-yl)propan-2-yl]carbamate: Into a 5000-mL 4-necked round-bottom flask purged and maintained with an inert atmosphere of nitrogen, was placed a solution of 2-(2-chloro-6-methylpyridin-4-yl)propan-2-amine (175 g, 948 mmol) in dioxane (3.5 L), di-*tert*-butyl dicarbonate (207 g, 950 mmol, 1.0 equiv). The mixture was heated to 95 °C for 1 hour, cooled to ambient temperature, diluted with toluene (2 L), and concentrated *in vacuo*. The residue was subjected to silica gel chromatography (eluting with ethyl acetate/petroleum ether; 1:10), providing the titled compound (142 g. 53% for two steps) as a white solid.

Preparation of 4-(2-[[(*tert*-butoxy)carbonyl]amino]propan-2-yl)-6-chloropyridine-2-carboxylic acid: Into a 5-L 4-necked round-bottom flask purged and maintained with an inert atmosphere of nitrogen, was placed a solution of *tert*-butyl *N*-[2-(2-chloro-6-methylpyridin-4-yl)propan-2-yl]carbamate (140 g, 492 mmol) in a mixture of water and *tert*-BuOH (2/1 L) and potassium permanganate (621 g, 3.93 mol, 8.0 equiv). The resulting mixture was heated at 95˚C for 6 hours, cooled to ambient temperature, and treated with *iso*-propanol (1g). The mixture was stirred for 18 hours, the solids were filtered off, and the pH of the filtrate was adjusted to 2 with aqueous HCl. The resulting mixture was extracted with ethyl acetate (3 x 3 L) and the combined organic extracts were washed once with brine (3 L), dried with magnesium sulfate, filtered, and concentrated *in vacuo*, providing the titled compound (120 g, 78 % yield) as a white solid.

Preparation of methyl 4-(2-[[(*tert*-butoxy)carbonyl]amino]propan-2-yl)-6-chloropyridine-2-carboxylate: Into a 3000 mL 3-necked round-bottom flask purged and maintained with an inert atmosphere of nitrogen, was placed a solution of 4-(2-[[(*tert*-butoxy)carbonyl]amino]propan-2-yl)-6-chloropyridine-2-carboxylic acid (120 g, 381 mmol) in methanol : dichloromethane (600 mL : 600 mL). This was followed by the addition of (trimethylsilyl)diazomethane (381 mL, 2.0 M in hexanes, 2.0 equiv) dropwise with stirring at 0˚C. After stirring for 1 hour, nitrogen gas was bubbled through the mixture for 30 minutes. Toluene was added (1.0 g, 10 mL) and the mixture was concentrated *in vacuo*. The residue was subjected to silica gel column chromatography (eluting with ethyl acetate: petroleum ether; 1:10), providing the titled compound (100 g, 80 % yield) as a white solid; low resolution mass spectrometry (ES+) m/z 328 $(M+H)^+$. ^{1}H-NMR (300 MHz, DMSO-d^6): δ 7.93 (d, J = 1.5 Hz, 1H), 7.82 (s, 1H), 7.61 (d, J = 1.5 Hz, 1H), 7.52 (s, 1H), 3.85 (s, 3H), 1.47 (s, 6H), 1.30 (s, 9H) ppm.

Preparation of methyl 4-(2-((tert-butoxycarbonyl)amino)propan-2-yl)-6-(4-fluorophenyl)picolinate: methyl 4-(2-((*tert*-butoxycarbonyl)amino)propan-2-yl)-6-chloropicolinate (26.3 g, 80 mmol), (4-fluorophenyl)boronic acid (16.8 g, 120 mmol), cesium carbonate (35 g, 107 mmol) and [1,1'-bis(diphenylphosphino)ferrocene]dichloropalladium(II) (2.93 g, 4.00 mmol) were taken up in toluene (400 mL) and water (40 mL). Nitrogen gas was bubbled through the solution for 10 minutes before the flask was heated at 85 °C for 1 hour. The mixture was cooled to ambient temperature, diluted with saturated aqueous sodium bicarbonate

(500 mL) and extracted with ethyl acetate (2 X 500 mL). The combined organic extracts were washed once with brine (500 mL), dried with sodium sulfate, filtered, and concentrated *in vacuo*. The mixture was subjected to silica gel chromatography (RediSep-Rf-1500 g, 0-100% EtOAc/hexane, 12 column volumes) to provide the titled compound (29 g, 93 % yield) as a white solid.

Preparation of *tert*-butyl (2-(2-(4-fluorophenyl)-6-(hydrazinecarbonyl)pyridin-4-yl)propan-2-yl)carbamate: Hydrazine hydrate (55.1 mL, 1.13 mol) was added to a solution of methyl 4-(2-((*tert*-butoxycarbonyl)amino)propan-2-yl)-6-(4-fluorophenyl)picolinate (22.0 g, 56.6 mmol) in EtOH (566 ml). The mixture was heated at 100 °C for 1 hour, cooled to ambient temperature, and concentrated *in vacuo*. The residue was dissolved in ethanol (200 mL) and then concentrated *in vacuo*; the residue was then dissolved in ethanol (200 mL) and heptanes (200 mL), filtered, and then concentrated *in vacuo*. The residue was suspended in heptanes (200 mL), concentrated *in vacuo*, and dried in vacuo to provide the titled compound (22.0 g, 100% yield) as a white solid; low resolution mass spectrometry (ES+) m/z 389.3 $(M+H)^+$.

Preparation of *tert*-butyl (2-(2-(2-(4-cyclopropoxy-3-methoxybenzoyl)hydrazinecarbonyl)-6-(4-fluorophenyl)pyridin-4-yl)propan-2-yl)carbamate: To a solution of 4-cyclopropoxy-3-methoxybenzoic acid (4.25 g, 20.4 mmol) and ((3*H*-[1,2,3]triazolo[4,5-b]pyridin-3-yl)oxy)tris(dimethylamino)phosphonium hexafluorophosphate(V) (9.04 g, 20.4 mmol) in *N,N*-dimethylformamide (148 mL) was added *N,N*-diisopropylethylamine (16.2 ml, 93.0 mmol) and allowed to stir for 5 minutes before *tert*-butyl (2-(2-(4-fluorophenyl)-6-(hydrazinecarbonyl)pyridin-4-yl)propan-2-yl)carbamate (7.2 g, 18.5 mmol) was added. The mixture was poured into aqueous saturated sodium bicarbonate (250 mL) and extracted with ethyl acetate (2 x 500 mL). The combined organic extracts washed with brine (500 mL), dried with sodium sulfate, filtered, and concentrated *in vacuo*. The mixture was subjected to silica gel chromatography (RediSep-Rf-750 g, 0-75% ethyl acetate: hexane, 12 column volumes) to provide the titled compound (10.4 g, 97 % yield) as a white solid.

Preparation of MRK-1, 4-(2-aminopropan-2-yl)-*N'*-(4-cyclopropoxy-3-methoxybenzoyl)-6-(4-fluorophenyl)picolinohydrazide: *tert*-Butyl (2-(2-(2-(4-cyclopropoxy-3-

methoxybenzoyl)hydrazinecarbonyl)-6-(4-fluorophenyl)pyridin-4-yl)propan-2-yl)carbamate (2.1 g, 3.63 mmol) was dissolved in ethyl acetate (36 ml). Gaseous HCl was then bubbled through this solution for ~15 seconds before the vessel was sealed, allowed to stir for 30 minutes at room temperature, and then concentrated *in vacuo*. The resulting solid was filtered, washed with diethyl ether (3 X 100 mL) and dichloromethane (2 X 100 mL) and the solid was dried to provide the titled compound as a light yellow solid (1.7 g, 91 % yield); high resolution mass spectrometry (ES+) m/z 479-2116 $(M+H)^+$; [$(M+H)^+$ calculated for $C_{26}H_{28}FN_4O_4$, 479.2089]. ^1H NMR (600 MHz, DMSO) δ 8.27 – 8.23 (m, 2H), 8.12 (d, *J* = 1.7 Hz, 1H), 8.00 (d, *J* = 1.6 Hz, 1H), 7.49 (dd, *J* = 8.4, 2.0 Hz, 1H), 7.41 (d, *J* = 2.0 Hz, 1H), 7.31 (d, *J* = 8.4 Hz, 1H), 7.24 (t, *J* = 8.8 Hz, 2H), 3.80 (tt, *J* = 6.0, 2.9 Hz, 1H), 3.73 (s, 3H), 1.41 (s, 6H), 0.77 (dt, *J* = 7.3, 5.8 Hz, 2H), 0.66 – 0.60 (m, 2H) ppm. ^{13}C[27] NMR (150 MHz, DMSO) δ 166.4, 163.6, 163.1, 161.5, 154.7, 150.8, 150.1, 147.8, 133.8, 129.2, 124.8, 120.9, 119.4, 117.2, 115.5, 115.4, 112.9, 110.2, 55.2, 52.2, 51.5, 30.3, 5.7.

Preparation of ^{3}H-labeled MRK-1

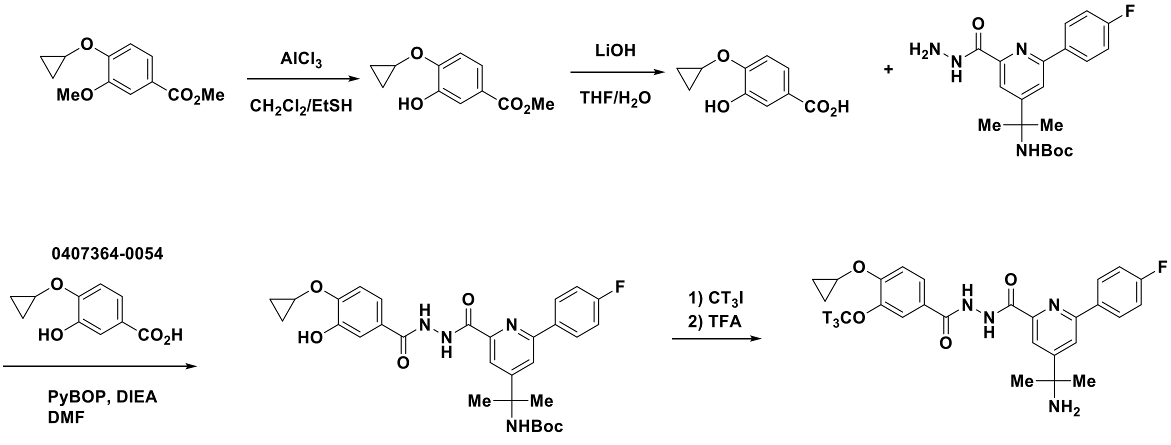

^{3}H-labelled MRK-1

Preparation of methyl 4-cyclopropoxy-3-hydroxybenzoate: Methyl 4-cyclopropoxy-3-methoxybenzoate (2.25 g, 10.12 mmol, *ACS Med. Chem. Lett.* **2017**, *8*, 1326−1330) was dissolved in dichloromethane (10 mL) and ethanethiol (10 mL). To this was added aluminum trichloride (4.05 g, 30.4 mmol) at 0 °C. The ice bath was removed, the mixture was warmed to room temperature, and after 30 minutes was diluted with water (100 mL) and extracted with dichloromethane (2 x 100 mL). The combined organic extracts were washed with brine (100 mL), dried with sodium sulfate, filtered, and concentrated *in vacuo*. The residue was subjected to silica gel chromatography (RediSep-Rf-80 g, 25-100% ethyl acetate in hexanes) to provide the titled compound (1.96 g, 93 % yield) as a white solid; low resolution mass spectrometry (ES+) m/z 209.2 $(M+H)^+$.

Preparation of 4-cyclopropoxy-3-hydroxybenzoic acid: Methyl 4-cyclopropoxy-3-hydroxybenzoate (100 mg, 0.480 mmol) was dissolved in tetrahydrofuran (4.8 mL) and methanol (4.8 mL). To this was added an aqueous solution of lithium hydroxide monohydrate (1.0 M aqueous, 4.8 mL, 4.80 mmol). The mixture was heated at 70 °C for 3 hours, cooled to ambient temperature, treated with HCl (5.7 µl, 5.76 mmol, 10 N). After stirring at room temperature for 20 minutes, the mixture was diluted with brine (25 mL) and extracted with dichloromethane (2 x 25 mL). The combined organic extracts were dried with sodium sulfate, filtered, and concentrated *in vacuo*, providing the titled compound (88 mg, 94 % yield) as a white solid; low resolution mass spectrometry (ES+) m/z 280.2 $(M+H)^+$.

Preparation of *tert*-butyl (2-(2-(2-(4-cyclopropoxy-3-hydroxybenzoyl)hydrazinecarbonyl)-6-(4-fluorophenyl)pyridin-4-yl)propan-2-yl)carbamate: To a solution of *tert*-butyl (2-(2-(4-fluorophenyl)-6-(hydrazinecarbonyl)pyridin-4-yl)propan-2-yl)carbamate (120 mg, 0.309 mmol), 4-cyclopropoxy-3-hydroxybenzoic acid (72.0 mg, 0.371 mmol) and ((3*H*-[1,2,3]triazolo[4,5-b]pyridin-3-yl)oxy)tris(dimethylamino)phosphonium hexafluorophosphate(V) (164 mg, 0.371 mmol) in *N,N*-dimethylformamide (2.5 mL) was added *N,N*-diisopropylethylamine (0.270 mL, 1.545 mmol). The mixture was stirred for 30 minutes, poured into aqueous saturated sodium bicarbonate (25 mL), and extracted with ethyl acetate (2 x 25 mL). The combined organic extracts were washed with brine (50 mL), dried with sodium sulfate, filtered, and concentrated *in vacuo*. The residue was subjected to silica gel chromatography (RediSep-Rf-24 g, 0-75% ethyl acetate in hexane), providing the titled compound (134 mg, 0.237 mmol, 77 % yield) as a white solid; low resolution mass spectrometry (ES+) m/z 565.2 $(M+H)^+$; ^1H NMR (600 MHz, DMSO-d^6) δ 10.73 (s, 1H), 10.38 (s, 1H), 8.52 (dd, J = 8.9, 5.8 Hz, 2H), 8.11 (d, J = 1.2 Hz, 1H), 8.00 (s, 1H), 7.49 (dd, J = 8.3, 2.2 Hz, 1H), 7.42 (d, J = 2.2 Hz, 1H), 7.39 (t, J = 8.9 Hz, 2H), 7.33 (d, J = 8.4 Hz, 1H), 1.62 (s, 6H), 1.38 (s, 9H), 0.86 − 0.84 (s, 2H), 0.76 − 0.74 (s, 2H) ppm.

Preparation of ^3H-labeled MRK-1, 4-(2-aminopropan-2-yl)-*N*'-(4-cyclopropoxy-3-(methoxy-*t*3)benzoyl)-6-(4-fluorophenyl)picolinohydrazide: tert-Butyl (2-(2-(2-(4-cyclopropoxy-3-hydroxybenzoyl)hydrazinecarbonyl)-6-(4-fluorophenyl)pyridin-4-yl)propan-2-yl)carbamate (0.5 mg) was added to a Tritium reaction vessel, followed by cesium carbonate (0.5 mg), DMF (0.1 ml), and [^3H]iodomethane (50 mCi). The vessel was sealed, and the solution was stirred overnight at room temperature. The reaction mixture was transferred to a 15 mL flask, and the vessel was rinsed with methanol (4 x 1 mL). The combined methanol washes were concentrated *in vacuo* and further purified by preparative HPLC. The purified desired intermediate fractions were combined and concentrated *in vacuo*. The residue was dissolved in dichloromethane (1 mL) and trifluoracetic acid (0.5 mL) was added. The mixture was stirred at ambient temperature for 2 hours and concentrated *in vacuo*. The final crude product was purified by preparative HPLC to give 3 mCi of the titled compound RSV Radiolabel MRK-1with radiochemical purity at 99% by HPLC. The ^3H-product coeluted with the unlabeled reference standard with HPLC. The specific activity of the product was 72.1 Ci/mmol determined by LC-MS.

Mass spectral data method: Specific activity was determined Mass Spectrometry, which was used in conjunction with the solution count to calculate mass concentration. The ^{3}H-labeled MRK-1tracer is 15.48% mono-tritiated, 18.20% di-tritiated, and 66.32% tri-tritiated and 0.00% unlabeled.

Analytical Data [Column: Ascentis Express C18, 3.6 x 100 mm, 2.7 µm @ 40 °C; Detection: UV, 210 nm; Injection Volume: 0.5 µL; Flow Rate: 1.8 mL/min; Run Time: 15 min; Mobile Phase: A: 0.1% formic acid in water, B: 0.1% formic acid in acetonitrile; Gradient A:B 90 :10 to 5: 95 over 13 minutes, hold at 5:95 for 2 minutes]

HPLC Purity for peak 5.50 minutes: 99.95%

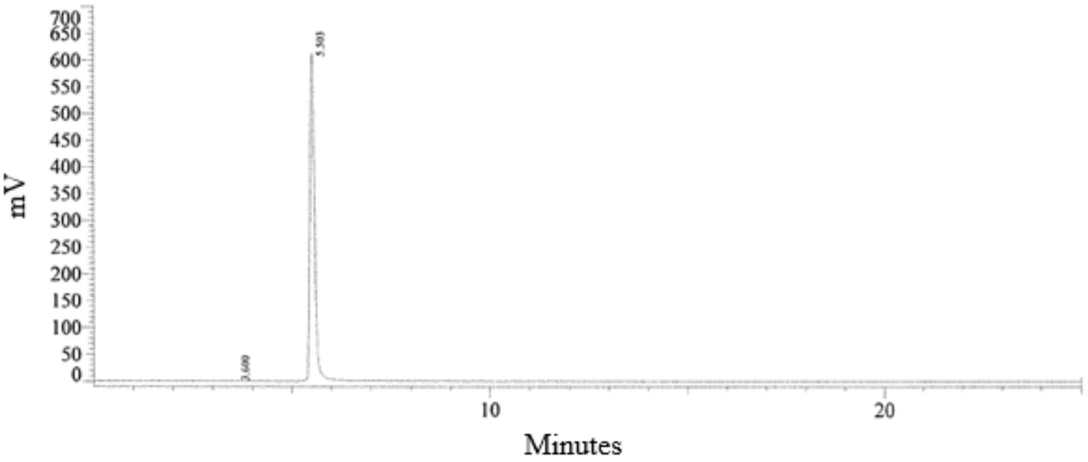

Supplementary Figure 12. Mass spectra and HPLC Analysis and Purity of MSD-RSV Radiolabel Tool

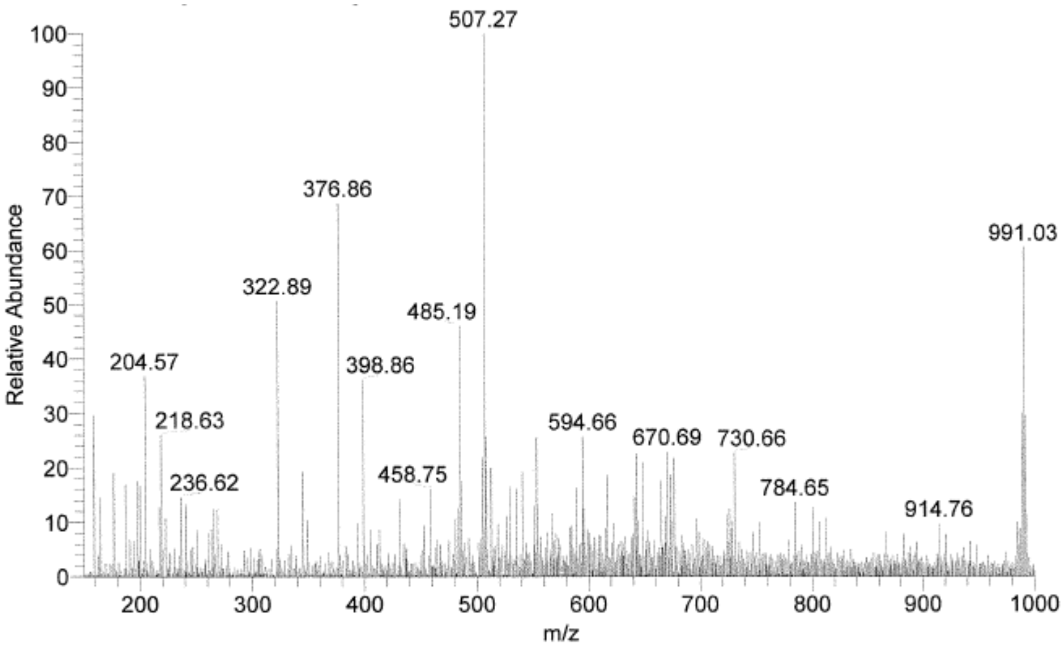

Supplementary Figure 13. Mass Spectra of ^{3}H-labeled MRK-1 tracer

Molecular Weight	[M+H]	# Tritium Atoms	Tracer Intensity	Reference Ion Intensity (%)	Net Tracer Intensity	Theoretical Ci/mmol	Measured Ci/mmol	Molecular Weight Calculation	Tracer % of Total (corrected)
478.520	479.14	0	0	100.00	0	0.00	0.0000	0.00	0.00
480.512	481.14	1	367722	0.00	367722	28.76	4.4519	74.38	15.48
482.504	483.14	2	432263	0.00	432263	57.52	10.4666	87.80	18.20
484.496	485.14	3	1575540	0.00	1575540	86.28	57.2242	321.34	66.32
486.488	487.14	4	0	0.00	0	115.04	0.0000	0.00	0.00
488.800	489.14	5	0	0.00	0	143.80	0.0000	0.00	0.00
490.472	491.14	6	0	0.00	0	172.56	0.0000	0.00	0.00
492.464	493.14	7	0	0.00	0	201.32	0.0000	0.00	0.00
494.456	495.14	8	0	0.00	0	230.08	0.0000	0.00	0.00
496.448	497.14	9	0	0.00	0	258.84	0.0000	0.00	0.00
Total			2375525		2375525				1.000000
						Measured Ci/mmol:			72.1428
						Molecular Weight:			483.517
						Specific Activity (mCi/mL):			149.2043
						Solution Count (mCi/mL):			1.020000
						Concentration (mg/mL):			0.00683626

Supplementary Table 3. Analysis of Percentage of Tritiation in ^{3}H-labeled MRK-1
