# Supplementary material for: Conserved allosteric inhibitory site on the respiratory syncytial virus and human metapneumovirus RNA-dependent RNA polymerases
Source: Commun Biol. 2023 Jun 19;6:649. doi: 10.1038/s42003-023-04990-0 (PMC10279679; doi:10.1038/s42003-023-04990-0)
Supplement: Supplementary file 4 — Reporting Summary [file 42003_2023_4990_MOESM4_ESM.pdf]

nature portfolio

Corresponding author(s): Rachel Fearns

Last updated by author(s): 5/19/2023

Reporting Summary

Nature Portfolio wishes to improve the reproducibility of the work that we publish. This form provides structure for consistency and transparency in reporting. For further information on Nature Portfolio policies, see our Editorial Policies and the Editorial Policy Checklist.

Statistics

For all statistical analyses, confirm that the following items are present in the figure legend, table legend, main text, or Methods section.

n/a	Confirmed	
☐	☒	The exact sample size (*n*) for each experimental group/condition, given as a discrete number and unit of measurement
☐	☒	A statement on whether measurements were taken from distinct samples or whether the same sample was measured repeatedly
☒	☐	The statistical test(s) used AND whether they are one- or two-sided<br>*Only common tests should be described solely by name; describe more complex techniques in the Methods section.*
☒	☐	A description of all covariates tested
☒	☐	A description of any assumptions or corrections, such as tests of normality and adjustment for multiple comparisons
☒	☐	A full description of the statistical parameters including central tendency (e.g. means) or other basic estimates (e.g. regression coefficient) AND variation (e.g. standard deviation) or associated estimates of uncertainty (e.g. confidence intervals)
☒	☐	For null hypothesis testing, the test statistic (e.g. *F*, *t*, *r*) with confidence intervals, effect sizes, degrees of freedom and *P* value noted<br>*Give P values as exact values whenever suitable.*
☒	☐	For Bayesian analysis, information on the choice of priors and Markov chain Monte Carlo settings
☒	☐	For hierarchical and complex designs, identification of the appropriate level for tests and full reporting of outcomes
☒	☐	Estimates of effect sizes (e.g. Cohen's *d*, Pearson's *r*), indicating how they were calculated

Our web collection on statistics for biologists contains articles on many of the points above.

Software and code

Policy information about availability of computer code

Data collection	EPU (Thermofisher) and Latitude S (Gatan) for RSV polymerase, and EPU for HMPV polymerase
Data analysis	Data analysis CryoSPARC (Structura Biotechnology) versions 2.15 for RSV polymerase and 3.3.1 for HMPV polymerase

For manuscripts utilizing custom algorithms or software that are central to the research but not yet described in published literature, software must be made available to editors and reviewers. We strongly encourage code deposition in a community repository (e.g. GitHub). See the Nature Portfolio guidelines for submitting code & software for further information.

Data

Policy information about availability of data

All manuscripts must include a data availability statement. This statement should provide the following information, where applicable:

- Accession codes, unique identifiers, or web links for publicly available datasets
- A description of any restrictions on data availability
- For clinical datasets or third party data, please ensure that the statement adheres to our policy

The maps including half-maps and the model coordinates of the two 3D-structures described in the manuscript have been deposited in the EMDB and PDB database with id EMD-29365 and 8FPI, respectively, for RSV polymerase; and EMD-29366 and 8FPJ, respectively, for HMPV polymerase. The data will be released to the public upon acceptance of the manuscript for publication.

Human research participants

Policy information about studies involving human research participants and Sex and Gender in Research.

Reporting on sex and gender	n/a
Population characteristics	n/a
Recruitment	n/a
Ethics oversight	n/a

Note that full information on the approval of the study protocol must also be provided in the manuscript.

Field-specific reporting

Please select the one below that is the best fit for your research. If you are not sure, read the appropriate sections before making your selection.

☒ Life sciences ☐ Behavioural & social sciences ☐ Ecological, evolutionary & environmental sciences

For a reference copy of the document with all sections, see nature.com/documents/nr-reporting-summary-flat.pdf

Life sciences study design

All studies must disclose on these points even when the disclosure is negative.

Sample size	No statistical methods were used to determine sample size.
Data exclusions	Quantification of some lanes of the acrylamide gels shown in Figure 4g were excluded because part of the samples were lost during phenol chloroform cleanup/ethanol precipitation and quantification would have been skewed. This is stated in the Figure legend.
Replication	All experiments were carried out with a least two to three independent biological replicates. The manuscript text states the number of biological and technical replicates in each experiment and what is meant by biological replicate.
Randomization	none
Blinding	none

Reporting for specific materials, systems and methods

We require information from authors about some types of materials, experimental systems and methods used in many studies. Here, indicate whether each material, system or method listed is relevant to your study. If you are not sure if a list item applies to your research, read the appropriate section before selecting a response.

Materials & experimental systems

n/a	Involved in the study	
☒	☐	Antibodies
☐	☒	Eukaryotic cell lines
☒	☐	Palaeontology and archaeology
☒	☐	Animals and other organisms
☒	☐	Clinical data
☒	☐	Dual use research of concern

Methods

n/a	Involved in the study	
☒	☐	ChIP-seq
☒	☐	Flow cytometry
☒	☐	MRI-based neuroimaging

Eukaryotic cell lines

Policy information about cell lines and Sex and Gender in Research

Cell line source(s)	HEp2 cells (human cervical carcinoma ATCC #CRL-1635) ; Calu-1 cells (human lung epidermoid carcinoma, ATCC HTB-54) ; Vero cells (African green monkey kidney cells ATCC #CRL-1587); Sf9 cells (Spodoptera frugiperda, Gibco, 11496015); Syrian baby hamster kidney (BHK)-21 cells engineered to express T7 RNA polymerase, provided by Dr. Karl Conzelmann (PMID:

	9847328).
Authentication	Not performed
Mycoplasma contamination	HEp-2, Calu-1 and Vero cells tested using MycoAlert Plus kit (Lonza, LT07-705). BSR-T7 cells tested with EZ-PCR™ Mycoplasma Detection Kit (Biological Industries, 20-700-20). All cell lines were negative for mycoplasma.
Commonly misidentified lines (See ICLAC register)	HEp-2 cells are widely used for experiments with RSV. They are known to be a HeLa cell contaminant and are listed as that by ATCC. Other cell lines used are not listed on the ICLAC register.
